# Beluga whale (*Delphinapterus leucas*) acoustic foraging behavior and applications for long term monitoring

**DOI:** 10.1371/journal.pone.0260485

**Published:** 2021-11-30

**Authors:** Manuel Castellote, Aran Mooney, Russel Andrews, Stacy Deruiter, Wu-Jung Lee, Megan Ferguson, Paul Wade

**Affiliations:** 1 Cooperative Institute for Climate, Ocean and Ecosystem Studies, University of Washington, Seattle, WA, United States of America; 2 Marine Mammal Laboratory, Alaska Fisheries Science Center, NOAA, National Marine Fisheries Service, Seattle, WA, United States of America; 3 Biology Department, Woods Hole Oceanographic Institution, Woods Hole, MA, United States of America; 4 Alaska SeaLife Center, Seward, AK, United States of America; 5 College of Fisheries and Ocean Sciences, University of Alaska, Fairbanks, Alaska, United States of America; 6 Department of Mathematics and Statistics, Calvin University, Grand Rapids, MI, United States of America; 7 Applied Physics laboratory, University of Washington, Seattle, WA, United States of America; Texas A&M University, UNITED STATES

## Abstract

Cook Inlet, Alaska, is home to an endangered and declining population of 279 belugas (*Delphinapterus leucas*). Recovery efforts highlight a paucity of basic ecological knowledge, impeding the correct assessment of threats and the development of recovery actions. In particular, information on diet and foraging habitat is very limited for this population. Passive acoustic monitoring has proven to be an efficient approach to monitor beluga distribution and seasonal occurrence. Identifying acoustic foraging behavior could help address the current gap in information on diet and foraging habitat. To address this conservation challenge, eight belugas from a comparative, healthy population in Bristol Bay, Alaska, were instrumented with a multi-sensor tag (DTAG), a satellite tag, and a stomach temperature transmitter in August 2014 and May 2016. DTAG deployments provided 129.6 hours of data including foraging and social behavioral states. A total of 68 echolocation click trains ending in terminal buzzes were identified during successful prey chasing and capture, as well as during social interactions. Of these, 37 click trains were successfully processed to measure inter-click intervals (ICI) and ICI trend in their buzzing section. Terminal buzzes with short ICI (minimum ICI <8.98 ms) and consistently decreasing ICI trend (ICI increment range <1.49 ms) were exclusively associated with feeding behavior. This dual metric was applied to acoustic data from one acoustic mooring within the Cook Inlet beluga critical habitat as an example of the application of detecting feeding in long-term passive acoustic monitoring data. This approach allowed description of the relationship between beluga presence, feeding occurrence, and the timing of spawning runs by different species of anadromous fish. Results reflected a clear preference for the Susitna River delta during eulachon (*Thaleichthys pacificus*), Chinook (*Oncorhynchus tshawytscha*), pink (*Oncorhynchus gorbuscha*), and coho (*Oncorhynchus kisutch*) salmon spawning run periods, with increased feeding occurrence at the peak of the Chinook and pink salmon runs.

## Introduction

The Cook Inlet beluga population was listed as endangered under the U.S. Endangered Species Act in 2008 after a rapid decline from 1300 animals in 1979 to 375 in 2008. The decrease was attributed to unregulated subsistence hunting [1]. The current estimated population size is between 250 and 317 belugas, with a median estimate of 279, and declining at a rate of -2.3% per year [2]. Unless the factors that are impeding recovery are determined and mitigated, this population may become extinct [3]. The Cook Inlet Beluga Recovery Plan [4] highlights a paucity of information on the belugas’ habitat use in Cook Inlet; information such as feeding grounds and diet are critically needed to establish a successful management strategy for the population, and thus promote recovery.

Because of their endangered status, Cook Inlet belugas are highly protected and thus invasive methods such as tagging are not currently permitted for studying feeding behavior. However, the Bristol Bay beluga population is estimated at 2000 to 3000 animals [5, 6]. This is the only U.S. non-migratory population, other than the Cook Inlet belugas or the highly cryptic and mostly unknown small population of belugas in Yakutat Bay, AK [7]. Bristol Bay beluga habitat is similar to Cook Inlet, largely restricted to an estuarine system characterized by large tidal fluctuations and turbid water. Their diet has been shown to be very similar, primarily salmon but also smelt, cod, and shrimp [8]. The acoustic environment of the two areas included in this study is also equivalent, predominated by natural noise sources from the strong tidal influence, and somewhat distant from the main anthropogenic noise sources such as commercial shipping in Cook Inlet and fishing vessels in Bristol Bay. Therefore, we consider Bristol Bay belugas a good surrogate for Cook Inlet belugas using animal-borne tags for exploring acoustic behavior when feeding.

Beluga whales have been described as one of the most vocal cetacean species [9]. They emit three types of social communication signals, narrowband frequency modulated tones termed whistles, broadband pulsed tones termed calls, and a combination of these two previous types, emitted simultaneously, termed mixed or combined calls [9, 10].These social signals range in frequency from approximately 200 Hz to 20 kHz and echolocation clicks extend upward of 120 kHz [9, 11]. The vocal repertoire of Cook Inlet belugas is not well known, only one descriptive study has been published so far [12], and no studies have been published for Bristol Bay belugas, but in other populations, social contexts have been related to highest vocal activity and feeding context to low or no vocal activity other than echolocation [13–16].

There is a growing body of literature focused on the acoustic characteristics of odontocete feeding behavior. The emission of echolocation buzzes directed at prey, termed terminal buzzes as they occur at the ending section of the click train [17, 18], has been used as an acoustic indicator of feeding occurrence in multiple species (e.g., harbor porpoise *Phocoena phocoena* and bottlenose dolphin *Tursiops truncatus* [19], Risso’s dolphins *Grampus griseus* [20]). However, buzzes are also known to occur during social interactions (e.g., Atlantic spotted dolphin *Stenella frontalis* and bottlenose dolphin[21], and spinner dolphin *Stenella longirostris* and Atlantic spotted dolphin [22]); therefore, it is important to characterize the acoustic properties of buzzes in both contexts to allow their discrimination (e.g., Marrero Pérez et al. [23] on short-finned pilot whale *Globicephala macrorhynchus*). Social and foraging buzzing in medium/large size odontocetes are often clearly distinguished by their position along the dive profile, with feeding occurring at the bottom or deep in the profile, while social interactions occur at or near the surface (e.g., Risso’s dolphin [20], short-finned pilot whale [23], killer whale *Orcinus orca* [24, 25]). In those species, this behavior allows for a selection of specific tag data periods to characterize foraging buzzes with low risk of confusion with social buzzes. However, acoustic behavior during the dive vertical profile has not yet been described for belugas. Both Bristol Bay and Cook Inlet beluga habitat have very similar depth ranges, rarely exceeding 100 m, and belugas in both populations are typically found nearshore [26, 27]. Although the dive data have yet to be summarized for Bristol Bay, dive depths in Cook Inlet average 4 m [28]; thus, depth separation of social and feeding buzzing from tag data is not applicable for these populations.

In the case of Cook Inlet belugas, passive acoustic monitoring is an effective way to monitor their seasonal occurrence year-round, and the temporal features of their echolocation signals have been used to describe feeding occurrence [29, 30]. This study aimed to improve our knowledge of the acoustic characteristics of beluga echolocation during feeding behavior in Bristol Bay belugas and develop methods for assessing Cook Inlet beluga prey preferences and feeding habitats based on passive acoustic monitoring. We identified feeding and social periods in Bristol Bay belugas based on stomach temperature sensing and acoustic behavior recorded in animal-borne tags. We characterized echolocation buzzes from both behavioral contexts. We then tested the identification of feeding occurrence in acoustic data from one mooring deployed in 2018 at a known foraging ground within Cook Inlet critical habitat. Finally, we described the relationships between the timing of spawning runs by different species of anadromous fish and beluga feeding occurrence.

## Material and methods

### Beluga whale capture location

Field work was part of the Bristol Bay Beluga Population Health Assessment Program, coordinated by the National Marine Fisheries Service, Alaska Department of Fish and Game, and Alaska SeaLife Center, following wild beluga capture and release procedures similar to those established in the 1990s [31–33]. Field work was based in Dillingham, AK, and involved launching small vessels to search for belugas in Nushagak Bay (west side of Bristol Bay). This study includes data from belugas temporarily restrained (up to 2 hours) for biological sampling and tagging in May 2014 and August 2016. All experimental work was conducted under National Marine Fisheries Service (NMFS) permit no. 14245 and in accordance with the Institutional Animal Care and Use Committee (IACUC) protocols of the Marine Mammal Laboratory of the Alaska Fisheries Science Center (MML/AFSC) (ID no. AFSC-NWSC2012-1 and AFSC/NWFC 2013–6), Woods Hole Oceanographic Institution (WHOI; ID no. BI166330), and the Alaska SeaLife Center (AUPs R12-08-07 and R16-04-04).

### Beluga whale instrumentation

Belugas were instrumented with three instruments: 1) an archival digital tag DTAG v3 [34] attached via four suction cups that continually recorded stereo sound, whale motion via tri-axial accelerometers and magnetometers, and depth; 2) a stomach temperature transmitter (STP v2, Wildlife Computers); and 3) a satellite-linked time-depth-recorder and STP receiver (SPLASH10-L-280B or SPLASH10-FL-238L Fastloc GPS, Wildlife Computers) that was attached via implanted nylon rods [31] near the anterior terminus of the dorsal ridge, providing the whale location, the dive depth and STP data to the Argos satellite system.

The DTAG was activated and placed by hand on the whale seconds before the animal’s release. To improve suction cup performance, the selected area of skin to deploy the DTAG was washed with a sponge and distilled water to remove any water-suspended glacial silt on the skin surface. The preferred area was as close as possible to the blowhole but behind the wrinkles formed in the neck area during head movements. DTAGs were deployed 12 to 33 cm from the blowhole (see Table 1), parallel to the longitudinal axis of the whale. Tags detached automatically following the pre-programmed release period. The drifting tag was located via its VHF beacon signal with support from aerial, land and vessel teams, and retrieved to download data.

**Table 1:** DTAG data sets and availability of concurrent stomach temperature (STP) or satellite location data for belugas instrumented during the Bristol Bay beluga population heath assessment program in 2014 and 2016.

Whale	DTAG data (h)	STP data	Satellite location data
**DLBB14-04**	18.5	No	Yes (ARGOS)
**DLBB14-05**	21.1	Yes	Yes (FastlocGPS)
**DLBB16-01**	5.8	Yes, but no feeding events	Yes (FastlocGPS)
**DLBB16-02**	23.9	No	Partial (4 FastlocGPS fixes)
**DLBB16-04**	34	Yes, but only 1 feeding event	Yes (ARGOS)
**DLBB16-06**	13	Yes, via recovery of tag	No
**DLBB16-09**	13.3	No	No

Results included in this report are highlighted in bold.

The STP was inserted into the stomach via a disinfected gastric tube. The STP was held in the end of the tube, which was inserted by opening the mouth of the restrained beluga, as with tube feeding or gastric lavage.

### Instrumentation settings

Three high frequency DTAG v3 were used in this study sampling sound at 240 kHz with a usable frequency response from 80 Hz to 120 kHz. Pressure sensor data and data from tri-axial accelerometers and magnetometers were sampled at 500 Hz per channel. One whale (DLBB16-04) was tagged with a prototype DTAG (s/n D416) capable of longer recording times due to extra memory and power. The STP was set to transmit temperature to the satellite-linked tag every 30 s at a 5 kHz carrier frequency.

The SPLASH10-L-280B tags were set to sample depth and water temperature at a rate of 1 Hz and summarize those data for transfer via the Argos system. Position estimates were calculated by the Argos system based on the Doppler shift of the Argos transmissions, which were scheduled to occur every 10 sec as long as the whale was at the surface. Argos position estimates were calculated using the Kalman filter method [35]. The SPLASH10-FL-238L Fastloc GPS tags recorded depth and temperature similarly, and although they used the Argos system for data relay, position estimates were based on GPS instead of Argos. These tags were set to attempt to collect a Fastloc GPS snapshot every 30 min as long as the whale was at the surface.

Both types of SPLASH tags received transmissions from the ingested STPs [36, 37]. The STPs transmitted stomach temperature every 30 sec, and the SPLASH tags decimated the stomach temperature (°C) readings to 1 every 2 minutes (low resolution mode, 0.5°C), for Argos transmission [37]. However, when triggered by a probable ingestion event, the tags summarized the STP data at 1-min intervals for transmission (high resolution mode, 0.1°C). High-resolution mode was triggered when either: 1) stomach temperature dropped below 33°C for at least two minutes, or 2) stomach temperature decreased at a rate of 0.8°C min^-1^ for two minutes. The STP data were recorded in high-resolution mode for two hours or when stomach temperature returned to within 0.5°C of the pre-ingestion temperature, whichever came first. Approximately 40 to 90 STP readings were compressed into a single Argos message, depending on the variability and rate of temperature changes. When a probable ingestion event was detected by the SPLASH tag, a “feeding event” message was generated that contained the time and duration of the event, the starting and minimum stomach temperatures during the event, the deepest depth reached during the event and the external water temperature at that time, all recorded at a 1-min sampling interval. Although the tags were programmed to transmit the STP, depth and GPS data multiple times, due to the limited surface time of belugas and the gaps in satellite coverage, some data messages may never be received.

### Bristol Bay beluga data analysis

Data from the DTAG’s motion and depth sensors were calibrated for temperature and orientation of the tag relative to the body axes of each tagged beluga, and were converted into depth, pitch, roll, and heading following methods described in Johnson and Tyack [34]. None of the datasets showed signs of changes in the position of the DTAG on the animal due to tag slippage; therefore, there was minimal need to correct for changes in orientation throughout the deployment.

**Fig 1:**
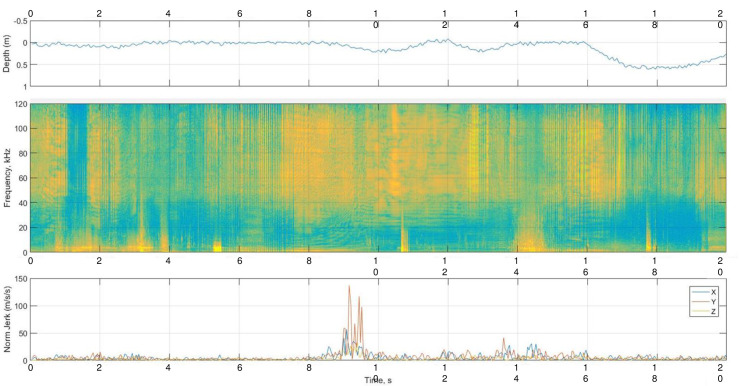
Screen interface of Matlab script tag3audit showing 20 s of concurrent data logged by the DTAG: depth (upper panel), spectrogram of sound (mid-panel), and tri-axial acceleration (lower panel) during chase and capture of prey by DLBB16-04. Buzzing starts at 7 s, just 1 s before the onset of an acceleration jerk at 9 s (presented as a breakdown of the 3 axes, pitch–X axis, roll–Y axis, and heading–Z axis, to highlight the Y axis contribution), while the whale was just below the surface. Time zero equates to 30.2 hrs since the whale was released, or 1:38 am AKDT on 16 May 2016.

Acoustic and sensor data analysis was carried out in MATLAB R2018a (MathWorks, Natick, MA, USA) using tag3audit and d3findclicks scripts from the DTAG toolbox (https://www.soundtags.org/DTAGs/DTAG-3/). The script tag3audit was used to manually scan and annotate each complete dataset by visualizing a spectrogram 20 s long (512 point Hanning window, 50% overlap), and its concurrent depth, pitch, roll, and heading (Fig 1). The annotation aimed to identify echolocation activity related to both feeding and social behaviors, in particular the emission of buzzes. To identify the behavioral context, feeding was presumed if stomach temperature drops indicative of ingestion of cold prey were present within the echolocation period. Stomach temperature drops often occurred during intense echolocation bouts including click trains containing terminal buzzes, and were associated with peaks in the rate of change of acceleration related to prey chasing (termed “jerk”, [18, 38, 39]. During these feeding periods, prey crunching sounds were searched for during the data annotation since there are precedents of these signals observed in DTAG acoustic data from other salmon eating odontocetes (i.e., [24, 40]). Any other signals related to the ending phase of the prey capture were also annotated, such as jaw claps. Buzzing periods related to a social context were identified as periods where the echolocating tagged whale was engaged in intense social calling and buzzing, in the absence of drops in stomach temperature. These social periods always occurred when other belugas were present in the acoustic data, identified by overlapping calls, whistles, and echolocation as well as high variation in the amplitude of these signals.

**Fig 2:**
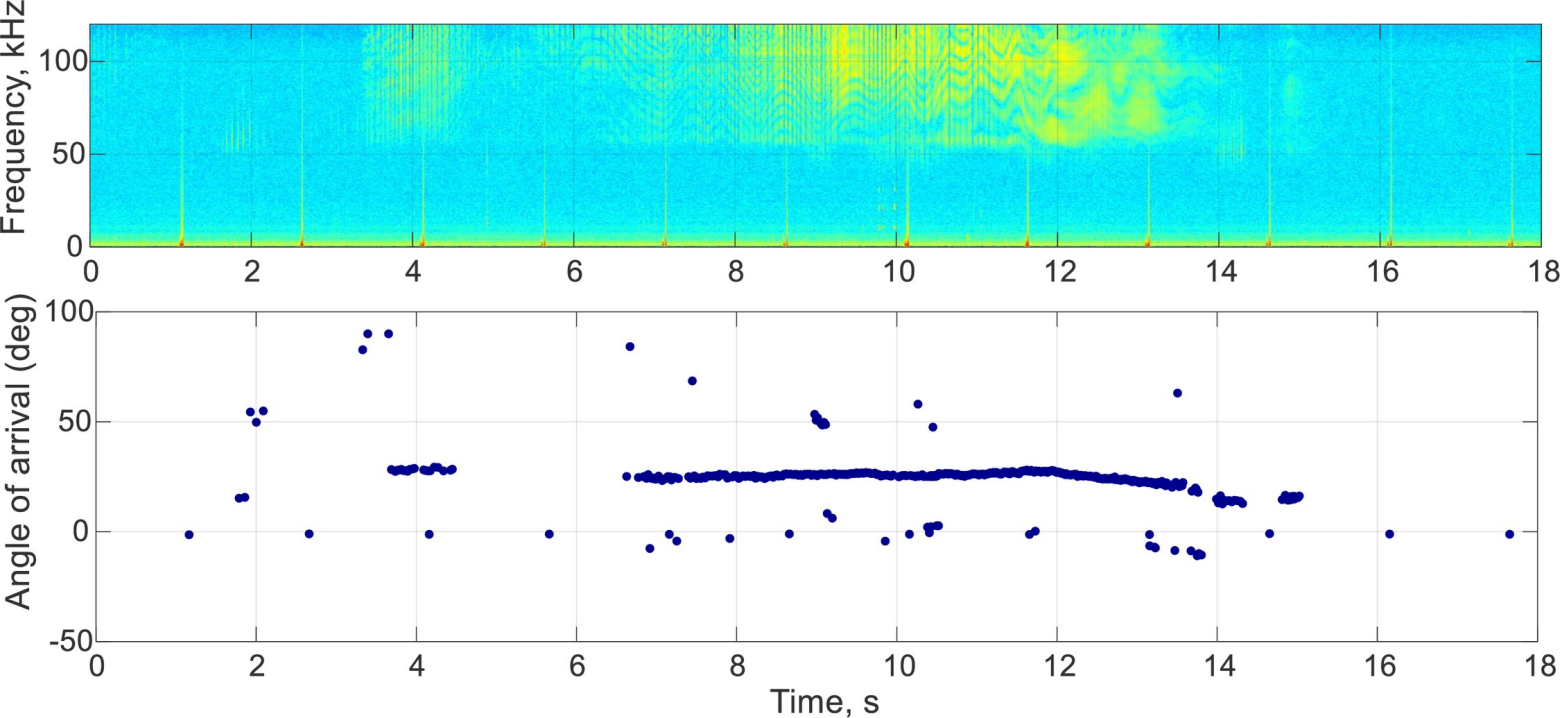
Spectrogram (upper panel) showing 18 s of data from DLBB16-04 emitting a feeding click train ending in a terminal buzz, corresponding angle of arrival of each click (horizontal blue line in the 20–25 degrees) measured with script d3findclicks from the DTAG toolbox. Time zero equates to 1.9 hrs since the whale was released, or 21:26 AKDT on 14 May 2016.

The script d3findclicks from the DTAG toolbox was used to scan feeding click trains with terminal buzzes and social buzzes produced by the tagged whales and selected during the annotation process to extract each click peak time, received level, and angle of arrival for the full event (the complete feeding click train ending in a terminal buzz, or the full social buzz). The script is a supervised energy detector following the techniques of Zimmer et al. [41] and Johnson et al. [42]. Before the analysis of all the selected data segments, click detection parameters were fine-tuned on a selection of click trains and buzzes with high signal-to-noise ratio (SNR) for highest efficiency. The script detects each click within a specified portion of acoustic data and extracts the peak time, angle of arrival and peak level in dB re 1μPa. These parameters are represented graphically to allow manual validation of each automatically detected click, as well as discrimination between clicks emitted by the tagged whale or close-by whales in the same group by assessing both the click amplitude and the increment in angle of arrival (Fig 2). False clicks (i.e., water splashing the tag at the surface) or click trains from nearby belugas were excluded from further processing and only clicks emitted by the tagged beluga were selected.

**Fig 3:**
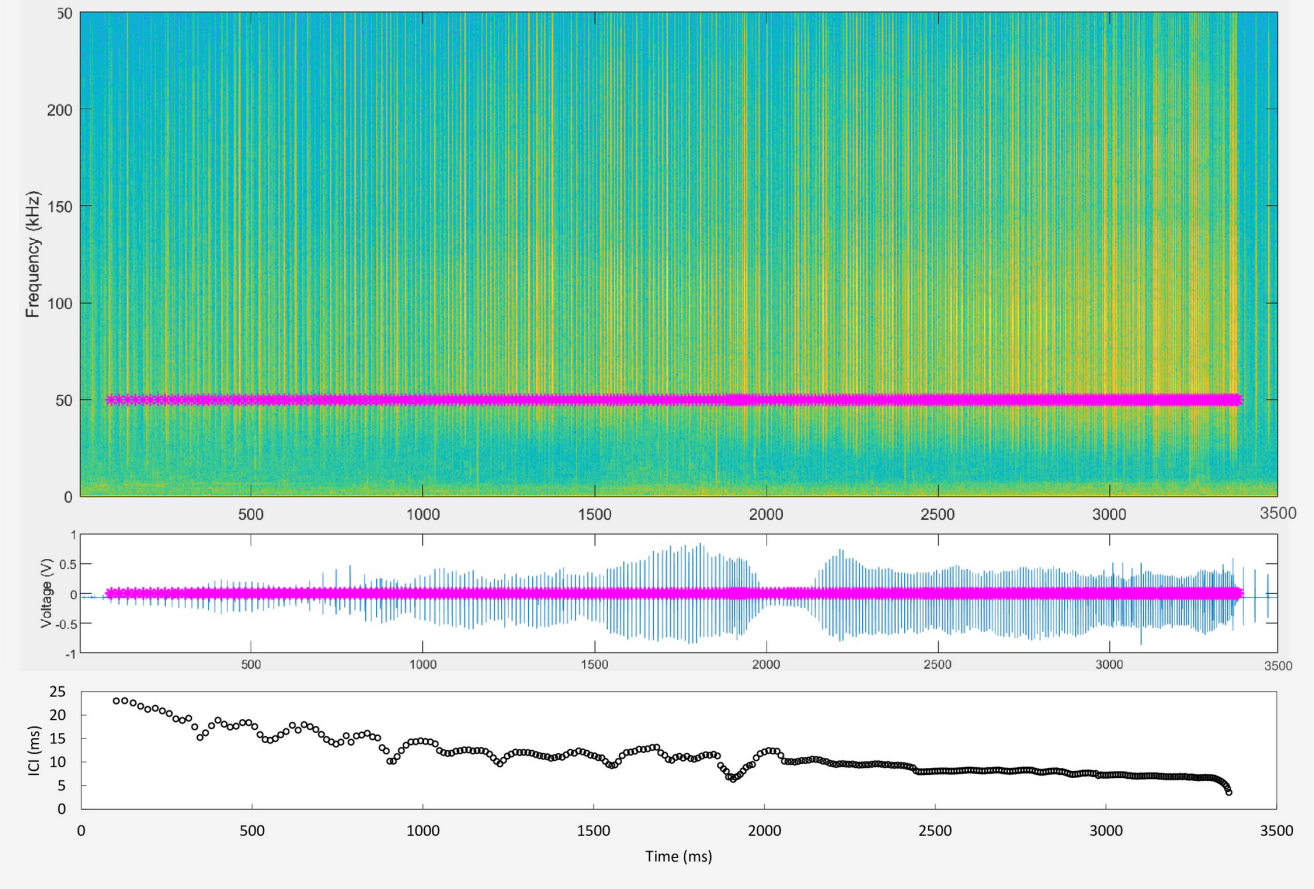
Spectrogram (upper panel) and waveform (middle panel) showing 3.5 s of data from DLBB14-05 emitting a feeding click train ending in a terminal buzz, and its corresponding inter-click interval (ICI) measured with script Callmark (lower panel). Purple asterisks mark each click peak time, with time zero equates to 14 hrs and 47 min since the beginning of the recording, or 5:08 am AKDT on 29 August 2014. Note clicks from another beluga were excluded at 700 to 900 ms and again at 2900 to 3500 ms.

The script d3findclicks was not efficient at extracting peak times of low SNR clicks occurring on buzzes, in particular on feeding terminal buzzes. Therefore, social and feeding buzzes were re-processed using the script Callmark [43] that allowed to successfully extract the peak times of low SNR clicks in buzzes. Callmark script runs a simple threshold detector on the signal waveform based on a threshold that is manually selected for each click sequence. The voltage time series are bandpass filtered in the frequency range 80 Hz to 120 kHz prior to click detection. A low highpass frequency is preferred, despite beluga click energy being absent below ~20 kHz, as it allows exploiting the vibrational component generated by the phonic system during click emission. All signals that exceed the threshold are logged and their peak times are calculated based on the waveform maxima within a 2 ms interval. False clicks, or clicks from other overlapping trains were identified by a lack of coherence in SPL and ICI, and were manually excluded from the selection for further analysis (see excluded clicks in Fig 3).

For each measured feeding click train or social buzz, peak times of each click were exported to Microsoft Excel 2016 to calculate inter-click intervals (ICI, time difference between consecutive click peak times) to obtain the ICI range (ICIr) derived from the difference between the maximum and minimum ICI (maxICI and minICI), and slope, derived from the linear regression of all ICI values for each click train or social buzz. Initial exploration of ICIs and ICI trends in feeding click trains suggested that terminal buzzing typically started when ICI was at or below 9 ms (see Fig 3 for a typical onset of a buzz when ICI was at 9 ms). Based on this threshold for feeding click trains, mean buzz ICIs are about two to three times shorter than the mean pre-buzz ICIs, and in some cases up to five times shorter. In order to characterize the rate of change in ICI along the buzzes, ICI increments (difference between consecutive ICI) and the range of this increment (ICI increment range, ICIir, derived from the difference between maximum ICI range and minimum ICI range) were measured starting at the first ICI ≤ 9 ms, and all consecutive clicks in terminal buzzes associated with feeding. The 9 ms selection criterion for defining the start of a terminal buzz was not applicable in social buzzes as these signals were entirely composed of buzzing, thus the entire social buzz was processed to calculate ICI and ICIir.

Cluster analysis and classification trees were used to investigate patterns in this multivariate dataset, as described below. Analyses were conducted in R version 4.0.5 [44]. Cluster analysis [45] was used to search for groupings in the buzz data. Multiple cluster algorithms (S1 Table) were applied to the buzz data and the resulting clusters were evaluated based on the presumed behavior classification of each observation in the cluster. Partitioning around medoids (PAM) from R package cluster [46] was used to separate the data into two clusters (k = 2). In total, 16 PAM algorithms were run, corresponding to all combinations of the following: distance metric (Euclidean, squared Euclidean, or Manhattan), number of acoustic variables (two or five), and data scaling option (scaled or unscaled). Preliminary investigations suggested that feeding buzzes could be cleanly separated from socializing buzzes using either minICI or ICIir. Therefore, one set of PAM models was constructed using all five acoustic variables (minICI, maxICI, ICIr, ICIir, and slope), a second set was constructed from only minICI and ICIir (two variables per model), and a third set was constructed from only minICI or ICIir (one variable per model). Because the cluster solution may be sensitive to the distribution of each of the acoustic variables, one set of PAM models was constructed from scaled data that were transformed by centering (subtracting the mean of each variable from each observed value) and then dividing the centered value by the variable’s standard deviation; a second set of PAM models was constructed from unscaled (untransformed) data.

Agglomerative nesting was used to construct hierarchical clusters with the hclust function from R package stats [44]. In total, 48 hierarchical models were built from a combination of two distance metrics (Euclidean and Manhattan), two (min ICI and ICI increment range) or five variables, seven linkage criteria, and either scaled or unscaled data (S1 Table). The linkage criteria were complete, single, average, weighted average, centroid, median, and Ward’s minimum variance method (see Kaufman and Rousseeuw 1990 for comprehensive details about each linkage method). Both distance metrics were investigated for the models built using complete, single, average, and weighted average linkage criteria. The centroid models used only squared Euclidean distance. The median models used only Euclidean distance. One set of models was built using Ward’s method with Euclidean distance and another set of models was built using Ward’s method with squared Euclidean distance.

Cluster algorithm performance was assessed using the Fowlkes-Mallows Index [47] from R package dendextend [48]. The Fowlkes-Mallows Index for each cluster model compares the cluster assignments to the presumed behavior (feeding or socializing) of each observation. The index ranges from 0 to 1, with 1 corresponding to perfect segregation of observations into two clusters according to presumed behavior category. The hierarchical models were evaluated by evaluating cluster assignments when k = 2.

Cluster assignments resulting from the top-performing cluster algorithms were further investigated using a decision tree classifier in the rpart package (Recursive Partitioning and Regression Trees version 4.1–15, [49]). The classification tree helps summarize the values of the acoustic variables that characterize the clusters created in the cluster analysis. The decision tree algorithm considers cluster assignment as a function of the collection of acoustic variables incorporated into the cluster algorithm. The objective of the decision tree algorithm is to begin with all observations pooled and then sequentially partition the data using dichotomous branching to create nodes that are more pure than the previous step. At each node, all variables are considered candidates for dividing the data into two branches, although the final branching rule for the node is based on only a single variable.

### Cook Inlet acoustic data

**Fig 4:**
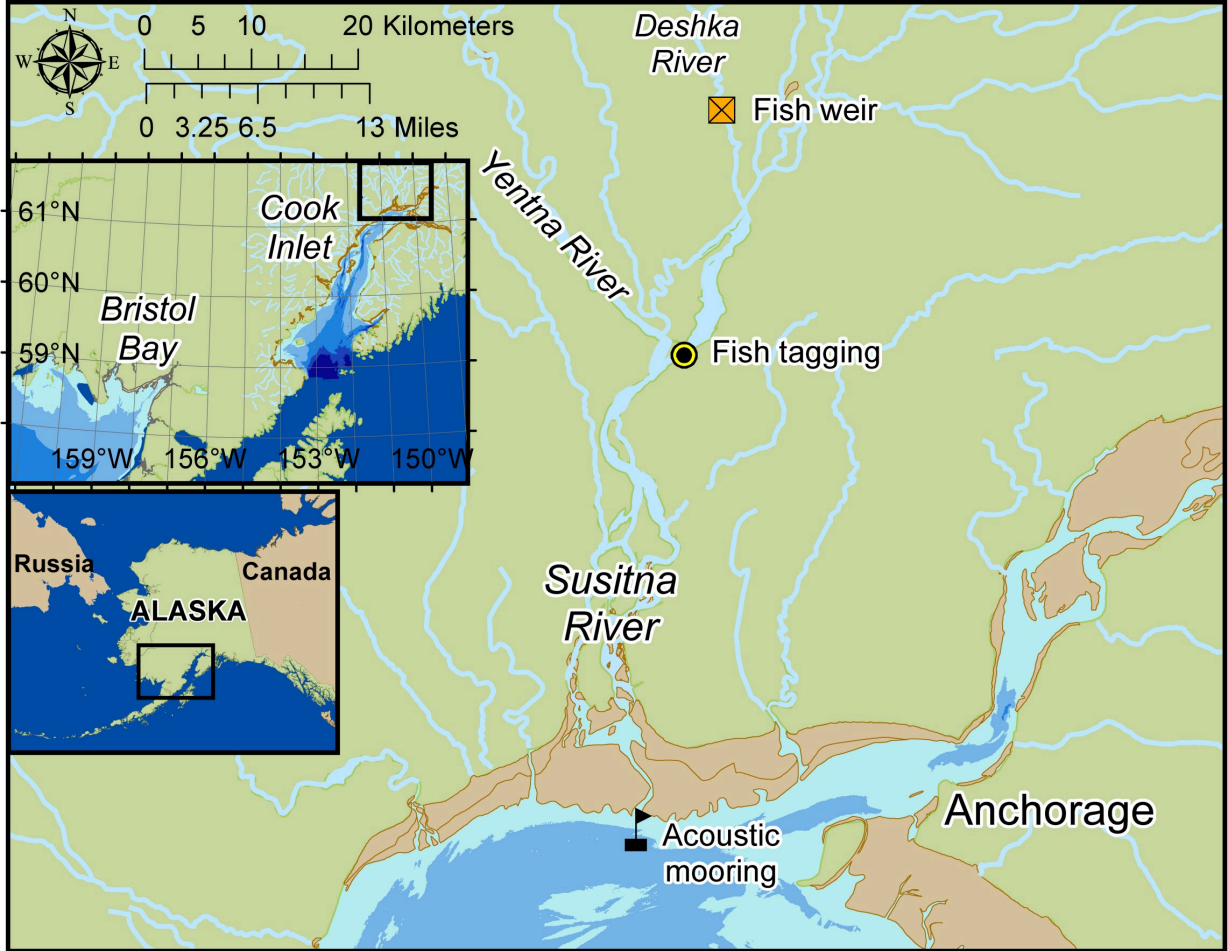
Map of upper Cook Inlet, depicting acoustic mooring location (black flag), fish tagging site and weir (circle and yellow box) at Deshka River (tributary of Susitna River). The map shows mud flats in brown (exposed at low tide), and 20-m depth contour (light to dark blue contour).

We used acoustic data from the Cook Inlet Beluga Acoustics (CIBA) research program, an ongoing long-term monitoring program initiated in 2008 in Cook Inlet, Alaska, within the critical habitat of the endangered Cook Inlet beluga population [29, 30, 50–53]. The mooring contained two instruments, a DSG-ST digital sound recorder (Loggerhead Instruments) that sampled the 0–12 kHz frequency range to detect beluga social signals, and a Cetacean and Porpoise Detector (C-POD, Chelonia Ltd.) that monitored the 20–160 kHz frequency range to detect beluga echolocation. The DSG-ST was programmed on a 33% duty cycle to prolong battery life, which resulted in recordings of 5 min every 15 min; the C-POD monitored continuously. The CIBA dataset selected to test our approach to detect beluga feeding behavior was from Susitna Delta mouth (Fig 4) from May to September 2018. This location has been described as one of the most important foraging grounds for this population during the ice-free season [53–55].

Because the ICIir was measured only in the feeding terminal buzz and not in the full length of the click train, the criteria for classifying feeding buzzes in C-POD mooring data was defined as a two-step process: First, identifying click trains with minICI < 8.976 ms; second, identifying those with ICIir < 1.49 ms to confirm the presence of a buzz in the click train. There is a small risk of finding fast ICIs derived from multiple individuals echolocating towards the C-POD simultaneously. However, the C-POD software processing includes algorithms specifically designed to minimize the chance of assigning overlapping click trains to a single source. The click train classifier uses the coherence of click features, the ICI of successive clicks, and the number, features and timing of intervening clicks to maintain independence of overlapping click trains [56]. The ICIir criterion is important as it avoids incorrectly classifying a click train with just one ICI under the minICI criterion. It also reduces the risk of overlapped click trains misclassified by the C-POD software as a single source matching the ICI criterion. This way, the detected section of the click train must be a buzz with ICI increments no larger than 1.49 ms, in which at least one ICI is below 8.976 ms in order to be classified as a feeding event.

Buzzes in C-POD mooring data with minimum ICI below 1 ms were omitted in the analysis, because multipath propagation of sound waves may result in double clicks due to different delays arriving at the C-POD along different paths; for example, by reflections from the water surface [57, 58].

### Cook Inlet beluga data analysis

Semi-automated detection of beluga social signals (i.e., calls and whistles) was obtained with a whistle and moan detector implemented in PAMGuard software version 2.00.14 beta (https://www.pamguard.org/), following the configuration detailed in Zhong et al. [59]. In summary, the whistle and moan detector implemented in PAMGuard was set up with a relatively low detection threshold (8 dB) to reduce the risk of missing beluga signals at the expense of triggering false detections (due to self-noise or anthropogenic noise). PAMGuard detection results were manually validated through visual and aural inspection of spectrograms in PAMGuard Viewer Mode, and labeled as either true or false detection. Analysis of C-POD data is described in detail in Castellote et al. [29]. In summary, C-POD data were analyzed using C-POD.exe v.2.043 (Chelonia Limited). All click train detections were manually validated by plotting the peak click frequency in the CPOD.exe analysis window with a time resolution of 100 ms. Cook Inlet beluga share their habitat with three other odontocete species, harbor porpoise (*Phocoean phocoeena*), Dall’s porposie (*Phocoenoides dalli*), and killer whale (*Orcinus orca*). As described in Castellote et al. [29], echolocation from these species can be manually classified in C-POD data based on considerable differences in peak frequency and click bandwidth. Minimum ICI from each validated click train was exported to Microsoft Excel 2016, and click detection times from click trains with minimum ICI ≤ 9 ms were also exported to calculate ICIir. We estimated beluga whale presence on an hourly basis. Specifically, any hour in which a beluga echolocation click train, call, or whistle was detected, by either data from DSG-ST or C-POD, was categorized as a detection positive hour (DPH). As such, a DPH could include a single type of beluga whale signal, or up to all three types (echolocation, calls, and whistles), and could include signals at different rates (e.g., one single call or many calls). This DPH approach reduced behavioral effects when quantifying beluga whale presence (e.g., avoided using number of signals detected as a metric of presence). The total number of DPH per day was used to calculate a 7-day running average.

Results from the Bristol Bay beluga echolocation classification tree analysis were applied to Cook Inlet C-POD data to identify click train buzzes considered to represent feeding behavior. Rather than using the absolute number of identified feeding buzzes to estimate foraging occurrence, each minute when at least one foraging click train was detected was classified as a foraging positive minute (FPM), and feeding occurrence was summarized as total FPM per day.

### Cook Inlet salmon count data and travel times

Pacific salmon escapement data were obtained from the Alaska Department of Fish and Game (ADF&G) online fish count data search tool accessible at https://www.adfg.alaska.gov/sf/FishCounts/. ADF&G operates river monitoring stations from about May through September. Data from all five species of Pacific salmon were searched for Susitna River. This river ends in an expansive delta with multiple channels. ADF&G is monitoring fish in Deshka River, a tributary of the Susitna mainstem, before the confluence of the tributary Yentna River; thus, the monitoring station at the Deshka River does not account for fish accessing the Yentna River or fish that continue their migration through the Susitna mainstem. The ADF&G weir station is located at Deshka river mile 7, approximately 50 river miles from the mouth of Susitna River (Fig 4). At this location, escapements of primarily pink, coho, and Chinook salmon are monitored using a resistance board weir.

Chinook salmon travel times for Susitna River have been previously documented [60, 61]. Data for 2018 have not been published, and were obtained from ADF&G (J. Campbell, 23 February 2021). Chinook salmon were tagged at the mainstem Susitna River marking site (river mile 34, Fig 4), from 22 May to 27 June 2018, and recaptured at the Deshka Weir (river mile 7). Travel time was calculated as number of days from tagging to recapture. Passive integrated transponder embedded dart tag (Model PDAT-PIT (HPT-12), Hallprint Australia) were anchored in the dorsal pterygiophores bones on the fish’s left side.

### Comparison of Cook Inlet beluga presence, feeding occurrence, and salmon runs

Daily counts of beluga DPH and FPM were compared to fish weir daily counts. Because fish that were around the mooring at the mouth of Susitna River took days to reach the river weir, Chinook salmon travel time was used to correct for this time lag. This approach assumes all salmon species have similar travel times, which is likely not the case. Unfortunately, only Chinook salmon travel time data are available for this river in 2018. Another limitation of this approach is the fact that travel time does not account for the distance from the mooring site to the salmon tagging site upriver. There is no information available on salmon travel time farther south than the tagging site for the Susitna River. Studies from other regions suggest travel speed is much higher during the initial sections of rivers where turbidity is high [62]; thus, application of the travel speed from the available data to this downriver section was not attempted. In order to account for the variability in salmon travel times from the tagging site to the fish weir farther upriver, individual travel times were used to compute a 5-day moving average from shortest to fastest travel time period. The smoothed travel time distribution was then used to calculate the predicted number of salmon at the tagging site based on the total counts at the weir. In order to identify the peak period of the spawning run for each salmon species, the 90^th^ percentile of predicted number of salmon per day at the tagging site was calculated. Dates with fish counts equal to or higher than the 90^th^ percentile defined the peak period of the spawning run for each salmon species. Total number of beluga FPM and DPH were calculated for the peak period of each spawning run.

## Results

### DTAG and STP datasets

A total of nine belugas were captured in August 2014, of which two were instrumented with DTAGs and satellite tags. Ten belugas were captured in May 2016, of which five were instrumented with DTAGs and satellite tags. Of these seven DTAG whales, only one from 2014, and two from 2016 provided concurrent data from the DTAG and both location and STP data from the satellite tag (Table 1). One of the successfully sampled whales in 2016 did not feed during the DTAG sampling period of 5 hrs and 50 min (DLBB16-01); therefore, data from only two whales (DLBB14-05 and DLBB16-04) were available to associate acoustic behavior, body movement, ingestion of prey and location. For the analysis presented here, we also included data from two more whales: DLBB16-02 for which we identified two feeding buzzes ending in prey crunching sounds, and DLBB16-06 for which we have DTAG and concurrent STP data, but no location data. This whale was found dead stranded four months after tagging, on 17 September 2016, from non-tagging related causes [63]. Although it was not transmitted via Argos, the STP data from the first 13 hours (when the DTAG was also attached to the whale) were retained in the tag’s memory archive, which was successfully offloaded.

### Bristol Bay beluga echolocation during feeding and social interactions

A total of 68 good quality feeding click trains containing buzzes and social buzzes were logged. Thirty-seven of these were correctly processed by the d3findclicks script to obtain received levels and angle of arrival (Table 2). Unfortunately, for the recording periods where echolocation bouts were identified and concurrent STP data indicated the tagged beluga was feeding, it was often difficult to isolate high SNR click trains including buzzes emitted by the tagged whale. This is in part because many click trains overlapped with echolocation from several close-by belugas or were masked by surfacing splashing noise (most feeding occurred in extremely shallow waters with the tag often breaking the surface). Thus, 31 of the 68 click trains with buzzes included large portions of the buzz sequence masked from splashing noise or hydrophones in the air, or overlapped with click trains from close-by belugas and attribution to the tagged whale was ambiguous due to high changes in angle of arrival (by head movement). Those 31 click trains were omitted from further analyses. An additional challenge was that the STP data were successfully transmitted via satellite on very few occasions. Thus, many click trains containing terminal buzzes from the tagged whales could not be related to stomach temperature and were not considered for this study. In total, 37 click trains with a total of 6767 ICIs were used for this study: 18 click trains with terminal buzzes containing 4135 ICIs during feeding context and 19 buzzes containing 2632 ICIs during social context.

**Table 2:** DTAG data sets with click trains (social buzzes or feeding click trains with terminal buzzes) associated to stomach temperature.

Whale ID	Feeding click trains		Social buzzes	
	Logged	ICI & ICIir measured	Logged	ICI & ICIir measured
DLBB14-05	4	2	6	3
DLBB16-02	2	2	0	0
DLBB16-04	3	1	9	6
DLBB16-06	26	13	18	10
Total	35	18	33	19

Each row indicates the number of click trains logged during the audit process, and the number of click trains that were successfully processed to obtain ICIs and ICIir during feeding and socializing contexts.

**Fig 5:**
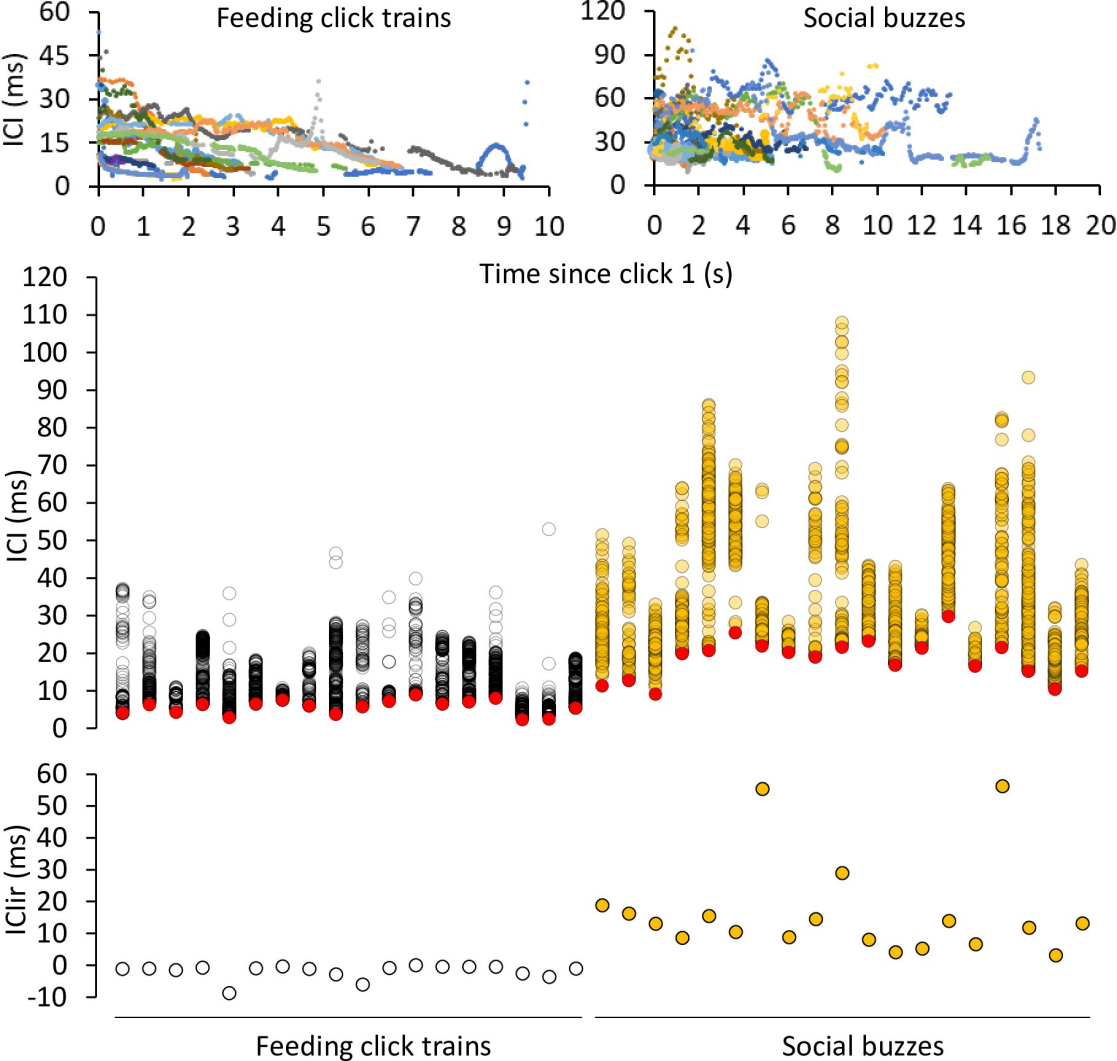
Upper panels—Inter-click interval (ICI) versus click time (starting at click 1 for each series) for feeding click trains (left, n = 18) and social buzzes (right, n = 19). Note Y- and X-axes differ between panels. Middle panel—Scatterplot showing the distribution of ICI (ms) per click train and their minICI (ms) in red, emitted during feeding behavior and social interaction. Lower panel—Scatterplot showing the distribution of ICIir (ms) per click train, emitted during feeding behavior and social interaction.

When looking at the ICI range and trend in click trains, feeding click trains typically decreased ICI gradually with minimum values during the final section of the terminal buzz. ICIs were distributed within a lower ICI range in feeding than in social buzzes. Social buzzes showed higher irregularity, higher ICI range, and longer duration (Fig 5). All feeding terminal buzzes presented lower minICI than social buzzes; lowest social ICI was 9.03 ms and highest minICI for feeding buzzes was 8.92 ms (Fig 5).

**Fig 6:**
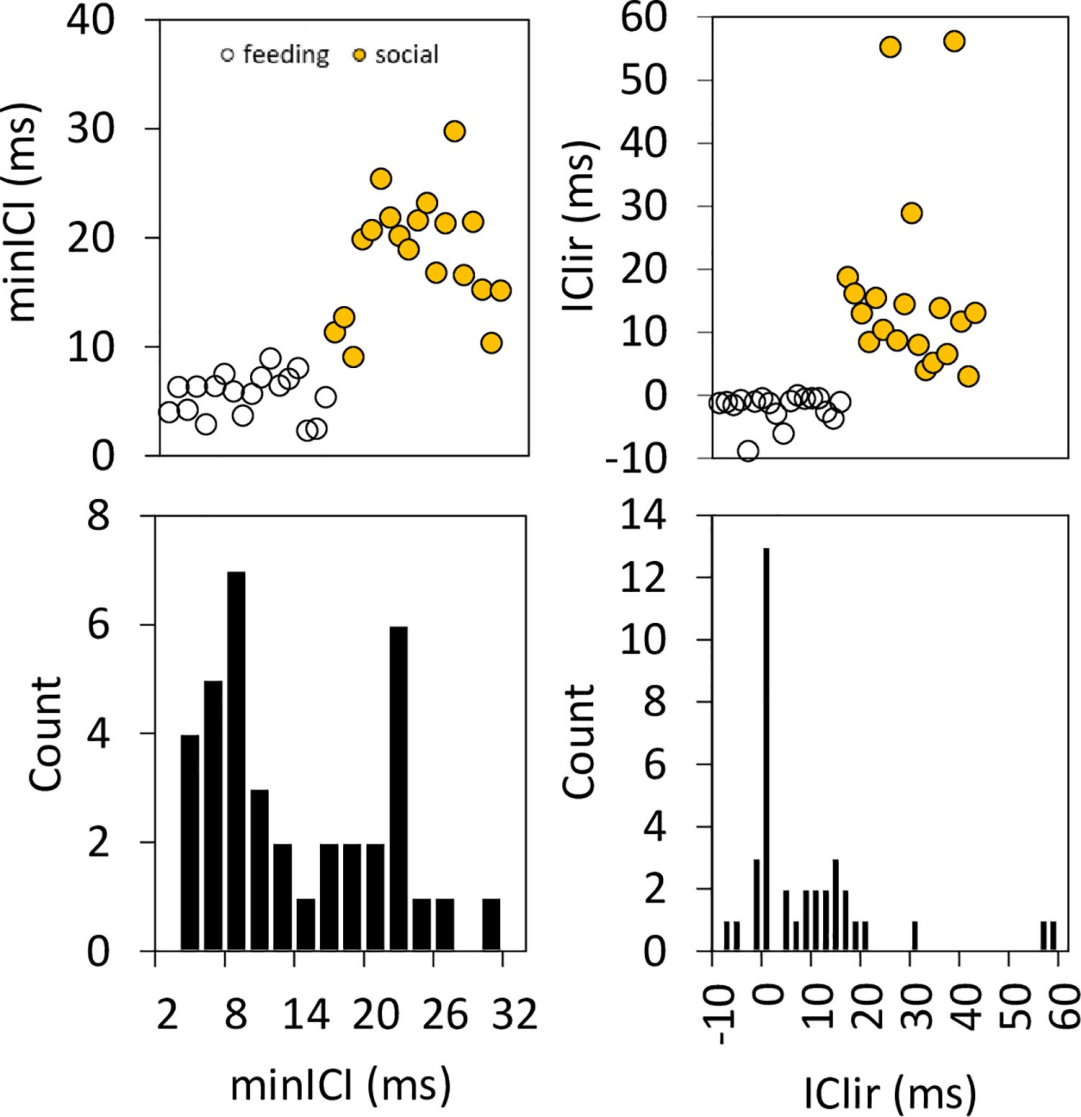
Scatterplot (upper panels) for minICI and ICIir from the 18 feeding click trains with terminal buzzes (yellow) and 19 social buzzes (black), and histograms (lower panels) with minICI and ICIir from all buzzes (feeding and social).

MinICI ranged from 2.3 to 8.92 ms in feeding click trains, and from 9.03 to 29.75 ms in social buzzes, with no overlap between the two. The overall distribution of ICI in feeding buzzes was highly skewed towards lower ICI, with a median of 11.79 ms. In contrast, ICI for social buzzes was more broadly distributed with median 32.25 ms. A histogram and the distribution of minICIs and ICIir for both types of click trains are presented in Fig 6. The minICI histogram shows a bimodal distribution, with a peak in the 7–8 ms bin that hosts the larger cluster for feeding terminal buzzes, and a second peak in the 21–22 ms bin for the larger cluster for social buzzes. The ICIir histogram shows a single peak in the -1-0 ms bin that hosts the larger cluster for feeding terminal buzzes, and a spread of cases from 3 to 20 ms for most of the social buzzes.

The ICI increments along the terminal buzzes of feeding click trains were more uniform than in social buzzes. Also, because the ICI gradually decreased along the feeding buzz, ICIir were all negative, from -8.82 to -0.42 ms, compared to positive increment ranges for social buzzes, from 2.98 to 18.72 ms (Figs 5 and 6). Considering these two variables, click train minICI, and buzz ICIir, echolocation click trains can easily be classified as feeding or social in context because no overlap in their ranges was observed.

**Fig 7:**
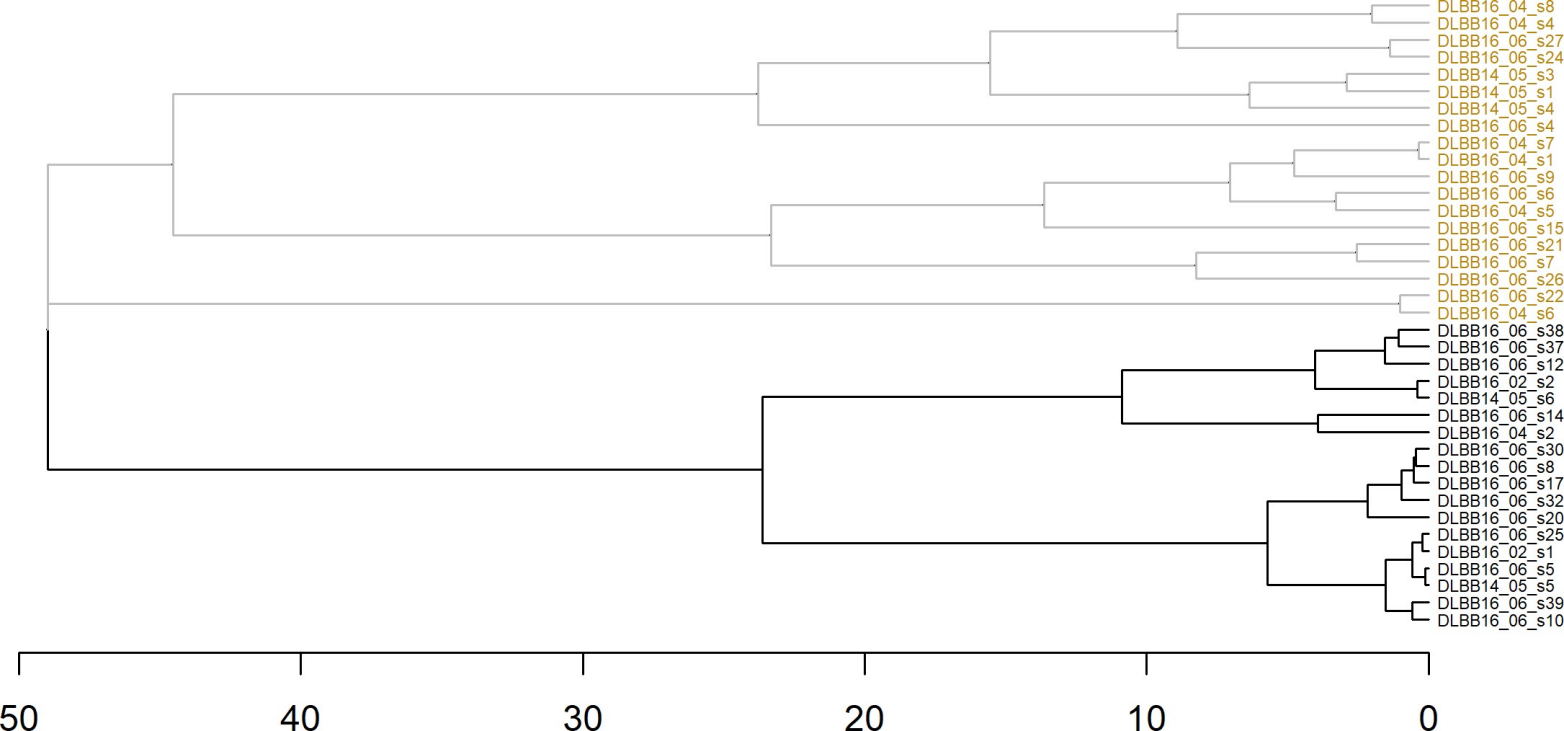
Dendrogram from the 2 top-performing cluster algorithms using agglomerative hierarchical methods, Euclidean distance, Ward’s minimum variance linkage criterion, unscaled data, and all 5 variables (minICI, maxICI, ICIr, ICIir, and slope) or 2 variables (minICI and ICIir) from a sample of 18 feeding click trains (black font color) and 19 social buzzes (yellow font color) from Bristol Bay beluga tagged with DTAG v3.

The cluster analysis resulted in models with Fowlkes-Mallows Index values ranging from 0.54 to 1.00 (S1 Table). Two cluster algorithms perfectly divided the buzzes according to presumed behavior. Both top-performing cluster algorithms used agglomerative hierarchical methods, Euclidean distance, Ward’s minimum variance linkage criterion, unscaled data, and generated the exact same clustering of buzzes in their dendrograms (Fig 7); one algorithm included all five acoustic variables and the other used only min ICI and ICI increment range (Fig 7). The best two partitioning-type cluster algorithms resulted in a single misclassified observation: Buzz DLBB16_06_s26 was included in the feeding cluster but DTAG and STP results suggest it was a social buzz. This buzz was closer in Euclidean distance to the feeding buzzes than to the social buzzes likely because of its ICIir (2.9 ms) was the lowest from all social buzzes, and its minICI was the second lowest (10.36 ms).

Identical classification trees were generated from the two top-performing cluster algorithms. The acoustic variable that the classification tree selected to assign buzzes to clusters was either minICI or ICIir, whichever variable was listed first among the explanatory variables in the model formulation. When minICI was listed first in the model formula, presumed feeding buzzes were cleanly separated from socializing buzzes, using minICI. Buzzes with minICI < 8.976 ms were classified as feeding and those with minICI ≥ 8.976 ms were classified as socializing. When ICIir was listed first, presumed feeding buzzes were cleanly separated from socializing buzzes, with ICIir < 1.49 ms classified as feeding and those ≥ 1.49 ms classified as socializing.

Prey capture events were often associated with strong side rolls, as seen in Fig 1 with a spike in the Y-axis jerk. Of the 37 feeding click trains, 27 (72.9%) ended with prey crunching sounds, similar to what has been described for killer whales predating on Pacific salmon [24]. On four occasions, the terminal buzz ended in a clear jaw clap. Only three feeding click trains did not show any evident sound at the end of the terminal buzz, and one feeding click train was masked by splashing noise by the end of the terminal buzz and therefore any following sound was not observable in the acoustic data.

### Cook Inlet beluga and salmon presence, and feeding results

**Fig 8:**
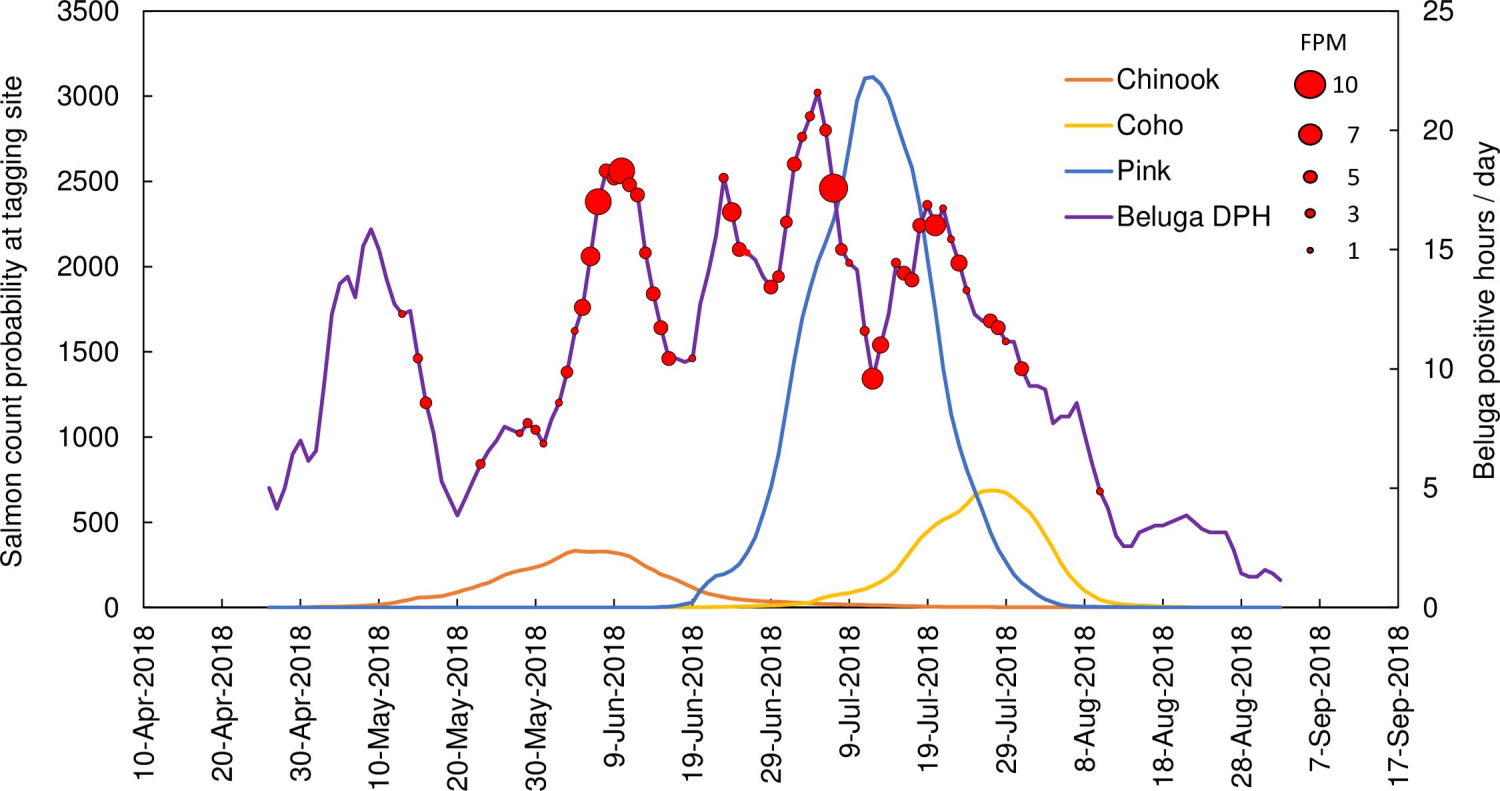
Predicted number of salmon per day at the tagging site at Susitna River for the five Pacific salmon species (fish/day), beluga daily presence in detection positive hours (DPH) per day, and feeding occurrence (FPM) per day (variably sized red filled circle). Note: chum and sockeye sustained very low predicted number of salmon per day at the tagging site and were omitted here.

Mooring dataset for 2018 ice-free season off Susitna Delta started on May 1^st^. Beluga whales were first detected on the same start date, and the daily presence was almost continuous until the end of the sampled period on September 9^th^. The daily trend in beluga presence peaked in early May and again in June to July (Fig 8). Feeding was detected on 56 days (Table 3).

**Table 3:** Number of days with feeding positive minutes (FPM) per month, and sum of FPM and non-FPM (belugas detected but no feeding) for those days off Susitna Delta in Cook Inlet, AK, during the 2018 ice-free season.

Month 2018	Days with FPM	Sum of FPM	Sum of non-FPM
May	8	14	78
June	21	89	692
July	26	87	816
August	1	1	34

Salmon runs were documented for all five species in Susitna River (weir at Deshka tributary). The Deshka tributary sustained large spawning runs of pink (58630), coho (12933) and Chinook (8544) salmon, and very few chum (131) and sockeye (74) salmon. Because of the low number of fish counts per day for these last two species, the predicted number of salmon per day at the tagging site was minimal (maximum of 6 for chum and 1 for sockeye); thus, these were omitted in Fig 8. However, chum salmon is known to be part of the diet of Cook Inlet beluga, and sockeye salmon is predated by beluga in other populations [8]. Of 1215 tagged Chinook salmon, 107 were recaptured at the weir between 4 June to 1 July 2018. Travel times ranged from 3 to 33 days with a median of 14 days (S1 Fig).

There is a good overlap in beluga presence, beluga feeding occurrence, and the peak of these salmon runs. Beluga feeding events were only detected within the salmon spawning run periods, with first detections at the onset of the Chinook run, on 13 May 2018, and last feeding events detected on 10 August 2018, when the coho run was already declining.

The Chinook salmon peak period overlapped with the second peak in beluga presence in early June. The pink salmon peak period fell in between the third and fourth peak in beluga presence in late June and mid-July but sustained the same number of total DPH that for the Chinook salmon peak (Table 4). The coho salmon peak period started on July 22^nd^, by the end of the last peak in beluga presence.

**Table 4:** Correspondence between the 90^th^ percentile of predicted number of salmon per day at the tagging site, start and end dates, beluga foraging positive minutes (FPM), and 7-day running average detection positive hours (DPH).

Salmon species	90^th^ percentile (fish count)	Date start	Date end	FPM	DPH (7-day average)
Chinook	270	2-Jun-18	12-Jun-18	55	163.9
Pink	2304	7-Jul-18	18-Jul-18	42	163.7
Coho	524	22-Jul-18	1-Aug-18	21	132.7

Chum and sockeye salmon runs sustained low fish counts and are not included in this table.

## Discussion

### Acoustic behavior of Bristol Bay beluga feeding

The DTAG audit process provided clear differences in the behavioral state of the tagged belugas. All seven datasets showed similar characteristics, with an initial “escape” mode after release, with no vocalizations or echolocation. These whales emitted a series of echolocation click trains immediately after release, likely in search of deeper waters and escape routes, but remained completely quiet for extended periods, on average 3.7 hrs, before echolocation was used again. No calls or whistles were emitted until a beluga group was rejoined, on average 5.6 hrs after release. It is surprising how these belugas navigated without the use of echolocation for such extended periods considering the extreme turbidity levels in Nushagak Bay, and the complexity of this shallow water, tidal-driven environment.

Three of the seven tagged whales resumed feeding before rejoining a beluga group, the rest did not feed until a group of belugas was joined. One tagged whale never joined other belugas in the 13.3 hrs of data obtained until tag release. For all the tagged whales, feeding behavior involved intense echolocation activity and little to no vocal activity. Feeding behavior was observed at very shallow depths (0–2 m) over mudflats only accessible at high tide periods. In few occasions, however, feeding behavior followed a more typical dive and surfacing sequence, where the whale would dive for short periods of 2 to 4 min at depths in the range of 3 to 10 m, in river channels or outside the coastal mud flats. However, intense echolocation occurred at both the surface and diving periods in contrast to other coastal odontocete foraging descriptions where echolocation related to feeding is exclusively detected at depth [40, 64]. Social behavioral state was typically identified by intense vocal activity at the surface and lack of any clear diving pattern. Both feeding and social contexts involved multiple animals around the tagged individual.

The results presented here are limited in sample size. While the instrumentation of seven belugas with DTAGs was unprecedented, the recordings only yielded a total of 68 good quality click trains containing buzzes under identifiable behavioral states (feeding or socializing), of which only 37 were successfully processed to obtain ICI characteristics of their buzzes. Three main limitations impeded obtaining a larger sample size. First, failure of the satellite tag to successfully uplink stomach temperature through the Argos system made acoustic data from three whales unusable for foraging analysis. The very low salinity due to river freshwater discharge in this area presented a challenge to the satellite tag’s system for sensing when it was at the surface. This system is based on a conductivity sensor and an adaptive algorithm, and transmissions during the first days were often not synchronized with the emergence of the antenna from the water. This problem has been identified in other studies in Bristol Bay [33]. Second, the tendency of Bristol Bay belugas to feed in groups and the lack of fused cervical vertebrae made the click train analysis challenging due to multiple concurrent echolocation bouts at varying angle of arrivals, including bouts from the tagged beluga varying up to +/-40 degrees from the longitudinal body axis. Third, tagged belugas spent most of their time feeding in extremely shallow waters (maximum dive depth was 12 m, median dive depth was 3 m) and splashing noise, related to surface water hitting the tag or other parts of the whale breaking the surface, as well as hydrophone exposure to the air, often masked echolocation signals or created signal gaps. These issues limited our ability to identify, log, and characterize the echolocation behavior in a substantial portion of the data.

Despite these problems, the fully analyzed 18 feeding click trains with terminal buzzes and 17 social buzzes yielded new data and obvious differences in their ICI distribution that are applicable to the identification of feeding behavior from acoustic data. Echolocation during foraging behavior was characterized by intense sequences often ending in buzzes. These terminal buzzes have been described in other odontocetes as well as bats [65, 66], and thus were expected to occur in our data. Amplitude of echolocation signals are highly range-dependent; this amplitude decreases with decreasing target range and is minimum in terminal buzzes [67–71]. This characteristic was also observed in beluga data and became a challenge when measuring click peak times with d3findclicks in terminal buzzes; it was the reason to switch to Callmark that allowed to manually lower the amplitude threshold in any given section of the waveform view when processing click trains.

The 18 feeding buzzes that we analyzed share a similar pattern: a gradual decrease in ICI until a minimum was reached at the end of the buzz. Unlike other odontocetes, beluga terminal buzzes ended at prey capture, often confirmed by crunch sounds, or at likely missed capture attempts often identified by a jaw clap at the end of the buzz sequence. Contrary to descriptions from trained beluga, none of the 27 feeding click trains with terminal buzzes and ending in prey crunches observed in our data continued after prey capture or lasted beyond the start of prey handling, and no vocal activity related to successful prey capture was observed (i.e., victory squeal; Ridgway et al. [72, 73]). Feeding terminal buzzes always ended by a prey crunch sound, a jaw clap, or a short period of silence until echolocation activity resumed.

Terminal buzz onset often presented a gradual decrease in ICI similar to terminal buzz descriptions in narwhals [74] but lacking the more typical drastic drop in ICI reported in other odontocetes [18, 75, 76]. Click rate at the onset of the buzzing in the click train was somewhat similar in social and feeding buzzes, but its progression along the buzz was quite distinctive. Feeding buzzes steadily declined in ICI reflecting the decrease in distance to the prey being chased, as described in other odontocetes [66]. This gradual decrease yielded very uniform ICI increments. However, social buzzes showed irregular variation in ICI rather than a patterned progression in ICI. This feature alone is distinctive; however, its application on acoustic data from moorings is limited, as only the ending section of click trains (i.e., the buzzing) would be distinctive. Because of the directional nature of beluga echolocation signals [77, 78], recorded click trains are incomplete and only short fragments corresponding to the whale aiming towards the general area of the mooring become available. Although exploring the ICI increment range in the short fragment of click train recording could be possible, this criterion alone might not allow discriminating a social buzz from a feeding buzz unless the terminal buzz is captured in the recording. There is a large margin of overlap in ICI between the two types of buzzes and only the shorter ICI observed in the ending part of the feeding buzzes would allow discriminating between these two behavioral states (Figs 5 and 6). Therefore, the two-step classification process described here, based on minICI and ICIir seems the most convenient approach to increase our capacity to identify beluga feeding buzzes from moored data based on the available DTAG results. Cluster analysis and classification tree results highlighted how these two criteria alone successfully grouped all feeding click trains with terminal buzzes and all social buzzes into two pure terminal nodes.

In our data, granting its limited sample size, there is no overlap in minimum ICI between feeding and social buzzes, although the lowest ICI in social buzzes is just 0.11 ms longer than the longest minimum ICI in feeding buzzes. This suggests that data from moored recorders will likely fail to identify feeding events unless the terminal buzz, or part of it, is captured in the recording. Therefore, it should be acknowledged that identifying feeding behavior based solely on echolocation features obtained from a mooring is a very restrictive approach. However, chances of detecting these particular signals will be higher in areas where belugas feed for prolonged periods near the mooring site, as described in Castellote et al. [30] for the Cook Inlet population, or as shown in the results of this study.

An important caveat, however, is that 13 feeding buzzes (72% of the sample size) come from one single beluga (DLBB16_06). If there are individual differences in the use of echolocation during foraging behavior, our results might not be representative of this population. We only successfully characterized five feeding buzzes in three other whales; however, both their minICI and ICIir fell within the variability observed in the 16 buzzes from whale DLBB16_06, and the 2 top-ranked dendrograms show how buzzes from this whale are widely distributed among all branches of the feeding cluster. It is also possible that the echolocation behavior during prey chase and capture might vary with prey type or the environment in which foraging occurs. Unfortunately, we were only able to characterize two feeding buzzes from the 2014 dataset that was obtained in August, compared to the 2016 dataset from May. Different anadromous fish species are targeted by Bristol Bay belugas in spring and summer [33]. The August 2014 feeding buzzes did not show any obvious differences in its spectral and temporal characteristics to feeding buzzes from May 2016, and the 2 top-ranked dendrograms show how the two 2014 buzzes are mixed with 2016 buzzes in the feeding cluster. Regardless, we would need to substantially expand our sampling efforts covering different periods of the year when belugas target different prey to confidently test for these hypotheses.

During prey chase and capture, the acceleration data often showed peaks in the Y-axis corresponding to a side roll (Fig 1). This behavior has not been described on belugas before but is seems common in other odontocetes where body tri-axial acceleration has been measured [38, 40, 64, 75, 79] as well as in mysticetes [80–83]. Side roll during feeding events on odontocetes has been hypothesized to increase echolocation performance, provide a more advantageous mouth position, or improve the use of vision for prey capture. A more likely explanation for side rolls on these tagged belugas is related to the extremely shallow nature of their foraging grounds, often only accessible during flooding tides. In this context, belugas swimming sideways would allow maximizing the fluking movement range as it would not be constrained by the bottom and water surface.

The characteristic odontocete acceleration jerk and increase in flow noise associated with final approach and prey capture, although evident in our data by an increase in masking at the low frequencies and clear audible change, were not as pronounced as in other mid-size odontocetes, based on the root-mean square values of the three-axis acceleration in ms^-2^ and the spectrograms shown in other publications [20, 23, 40, 84]. These differences could be caused, in part, by the location of the tag on the whale’s body, where most studies placed the tag on the dorsal section when the whales surfaced compared to a manual placement near the blowhole while the beluga was restrained. However, differences in the rate of whale movement during prey chase and capture are evident. Lower values in beluga jerks and flow noise reflect a slower swim speed during prey chase and capture. Beluga feeding mechanics have been described to be composed of discrete ram and strong suction components [85]. In contrast with other odontocetes where the ramming is more pronounced, belugas are able to purse the anterior lips to occlude lateral gape and form a small, circular anterior aperture, significantly enhancing suction generation. Suction generation in odontocetes is a function of hyolingual displacement and rapid jaw opening, and thus the lower jerk peaks observed in our data during prey chase and capture, rather than derived from acceleration from stronger fluking, could likely correspond to muscular activity from the jaw and hyolingual displacement for effective suction feeding.

### Cook Inlet beluga presence and feeding occurrence

The relationship between timing of salmon runs, beluga presence, and feeding occurrence should be interpreted with caution because of the unknown time lag between salmon presence in the mooring area and salmon captured at the tagging site at river mile 34. Safe minimum depth placed the acoustic mooring at a distance of ~5 mi from the river mouth (defined as the shoreline limit at the main channel in the Susitna Delta). That said, the general periods of higher presence of belugas, and dates when feeding was detected, can be compared to the general periods of peak spawning runs with the assumption that salmon passing through the mooring area are soon after (i.e., days) captured at the Susitna mainstem tagging site.

Beluga DPH followed an expected continued presence throughout the sampled period of May to September with two typical peak periods, one in spring and another in mid-summer, followed by a decreasing trend by late summer and early fall matching previous descriptions e.g., [30, 55, 86]. Belugas were detected almost every day from the first day of sampling in May. Feeding events detected at the mooring fell within the onset of the first salmon run of the season (Chinook), and the end of the last salmon run (coho) (Fig 8), and feeding was not detected in April or in August in absence of salmon running. Furthermore, highest number of FPMs occurred at the peak periods of two of the three salmon runs (Chinook and pink salmon). The coherent relationship between feeding occurrence and salmon run timing observed in the Susitna Delta during the 2018 season suggests the methodology applied to acoustically identify feeding events in mooring data is promising.

A peak in beluga presence occurred during the first two weeks of May before the first salmon spawning run of the season. Eulachon has been described as a likely important prey for belugas in spring (NMFS 2008). Eulachon begin returning to spawning areas in upper Cook Inlet, with the Susitna River as one of the most important watersheds, from mid-May to mid-June and return in quantities large enough to support a limited commercial fishery. The most probable total biomass in 2016 was estimated at 48,000 metric tons for the Susitna watershed [87]. Due to their high densities and lipid content, eulachon is likely a key food source for Cook Inlet beluga during this period when energy reserves are low and salmon presence in upper Cook Inlet is very limited. The peak in beluga presence detected in May in the Susitna Delta mooring dataset is likely a reflection of beluga exploiting this resource early in the ice-free season, supporting the key importance of eulachon as part of their diet. However, there are other known resident species of fish in the Susitna River that could be available for beluga in May, such as grayling *Thymallus thymallus*, burbot *Lota lota*, northern pike *Ptychocheilus oregonensis*, and rainbow trout *Oncorhynchus mykiss* [88]. Unfortunately, little information is available about their seasonality and biomass in this river, or the importance of these species in the diet of beluga populations.

Later in the season, Deshka River sustained a salmon run for Chinook, coho, and pink salmon, and small numbers of chum and sockeye salmon were also counted at the weir. The highest DPH in May occurred on May 9^th^, at the presumed peak of typical eulachon runs for this river [89]. However, feeding events were not detected until May 13^th^, when the Chinook salmon run was already ongoing, and these detections continued until the end of the last salmon run of the season in August. The marked temporality in feeding occurrence suggests that feeding behavior targeted at eulachon does not occur within the detection range around the mooring; in contrast, salmon feeding occurs around the mooring for the three main spawning runs sustained by the Deshka River. These differences in feeding behavior could be related to accessibility of prey. Perhaps scattered salmon are accessible around the mooring but eulachon, because they are smaller fish and lack a swim bladder [90], need to be in more concentrated densities to allow for an effective backscatter from beluga echolocation emissions. Such high densities have been observed inside river channels [88], but they might not occur near the mooring.

The peak of the Chinook salmon run predicted for the tagging site perfectly matched the second peak in beluga presence, from the 5^th^ to the 11^th^ of June, and sustained the highest number of feeding events (Table 4). The decrease in beluga presence on following days matched the decrease in Chinook salmon predicted counts, reaching a minimum by June 18^th^. This decrease in beluga presence was immediately followed by the onset of the pink salmon run and increase in beluga presence. The pink salmon run was the strongest of all and the one lasting longer at high counts per day. Interestingly, beluga presence varied considerably during this period but remained higher than 10 DPH/day, and overall presence during the Pink run peak period was almost identical to the one for Chinook salmon (Table 4). Feeding events occurred throughout the whole pink run, but the peak sustained the highest number of feeding events. The coho run peaked by 26-28^th^ of July, while beluga presence gradually decreased (but remained above 10 DPH/day); however, feeding events still occurred within this period.

Overall, these results reflect consistent presence in this area during the eulachon and the three salmon run periods sustained by the Deshka River, with increased feeding occurrence at the peak of the Chinook and pink salmon runs. The slight lack of synchrony observed between beluga presence, and the pink and coho salmon peak runs could be caused by differences in travel times across salmon species because only Chinook data were available. In this regard, it should be noted that the peak in beluga presence on July 5^th^ and July 19^th^ occurred precisely seven days earlier than the peak in predicted pink and coho salmon counts at the tagging site, respectively. Adding seven days of travel time to these two salmon species would maximize the correlation with beluga peak presence.

## Conclusions

A successful method has been developed based on acoustic tagging data of limited sample size to identify beluga feeding occurrence in passive acoustic data from moored instruments. This methodology can likely be refined once more tagging data becomes available, but its current application to passive acoustic data from a Cook Inlet beluga foraging ground showed promising results. The analyses presented here characterized the relationship between beluga feeding occurrence and the timing of salmon and eulachon spawning runs based on escapement data. Beluga presence and the timing of feeding events reflect a clear preference for the mouth of the Susitna River during the spawning runs by eulachon, Chinook, pink and coho salmon. The description of this relationship can likely be improved if more accurate data on salmon and eulachon travel times are obtained.

## Supporting information

S1 Fig Histogram of Chinook salmon 2018 travel times from the tagging site at the Susitna River mainstem (river mile 34) to the tributary Deshka River weir (river mile 7). (TIF)

S1 Table Table with the 48 cluster algorithms applied to the buzz data in descending Fowlkes-Mallows index rating. (XLSX)

## References

[1] NMFS. Conservation plan for the Cook Inlet beluga whale (Delphinapterus leucas). NOAA National Marine Fisheries Service, Juneau, AK. 2008. Available from: https://repository.library.noaa.gov/view/noaa/18275

[2] Shelden KEW, Wade PR. Aerial surveys, distribution, abundance, and trend of belugas (Delphinapterus leucas) in Cook Inlet, Alaska, June 2018. AFSC Processed Report 2019–09, Alaska Fisheries Science Center, Seattle, WA. 2009. Available from: https://apps-afsc.fisheries.noaa.gov/documents/PR2019-09.pdf

[3] Hobbs RC, Laidre KL, Vos DJ, Mahoney BA, Eagleton M. Movements and area use of belugas, Delphinapterus leucas, in a subarctic Alaskan estuary. Arctic 2005; 58(4):331–340.

[4] NMFS. Recovery plan for the Cook Inlet beluga whale (Delphinapterus leucas). NOAA National Marine Fisheries Service, Juneau, AK. 2016. Available from: https://repository.library.noaa.gov/view/noaa/15979

[5] Allen BM, Angliss RP. Alaska marine mammal stock assessments, 2014. U.S. Dep. Commer., NOAA Tech. Memo. NMFS-AFSC-301, 304 p. 2015. Available from: https://repository.library.noaa.gov/view/noaa/4945

[6] Citta JJ, O’Corry-Crow G, Quakenbush LT, Bryan AL, Ferrer T, Olson MJ et al. Assessing the abundance of Bristol Bay belugas with genetic mark-recapture methods. Mar Mamm Sci 2018; 34(3):666–686.

[7] Lucey W, Henniger E, Abraham E, O’Corry-Crowe G, Stafford KM, Castellote M. Traditional knowledge and historical and opportunistic sightings of beluga whales in Yakutat Bay, Alaska 1938–2013. Mar Fish Rev 2015; 77(1):41–46.

[8] Quakenbush LT, Suydam RS, Bryan AL, Lowry LF, Frost KJ, Mahoney BA. Diet of beluga whales (Delphinapterus leucas) in Alaska from stomach contents, March–November. Mar Fish Rev 2015; 77:70–84.

[9] Sjare BL, Smith TG. The vocal repertoire of white whales, Delphinapterus leucas, summering in Cunningham Inlet, Northwest Territories. Can J Zool 1986; 64:407–415.

[10] Au WWL, Carder DA, Penner RH, Scronce BL. Demonstration of adaptation in beluga whale echolocation signals. Journal of the Acoustical Society of America 1985; 77:726–730. doi: 10.1121/1.392341 3973242

[11] Blevins-Manhard R, Atkinson S, Lammers M. Spatial and temporal patterns in the calling behavior of beluga whales, Delphinapterus leucas, in Cook Inlet, Alaska. Mar Mammal Sci 2016; 33:1–22.

[12] YaI Alekseeva, Panova EM, Bel’kovich VM. Behavioral and Acoustical Characteristics of the Reproductive Gathering of Beluga Whales (Delphinapterus leucas) in the Vicinity of Myagostrov, Golyi Sosnovets, and Roganka Islands (Onega Bay, the White Sea). Biol Bull 2013; 40(3):307–317. 24171316

[13] Belikov RA Bel’kovich VM. Underwater Vocalization of the Beluga Whales (Delphinapterus leucas) in a Reproductive Gathering in Various Behavioral Situations. Oceanology 2003; 43(1):112–120.

[14] Karlsen J, Bisther A, Lydersen C, Haug T, Kovacs K. Summer vocalisations of adult male white whales (Delphinapterus leucas) in Svalbard, Norway. Polar Biol 2002; 25:808–817.

[15] Panova R.A. Belikov A.V. Agafonov V.M. Bel’kovich. The Relationship between the Behavioral Activity and the Underwater Vocalization of the Beluga Whale (Delphinapterus leucas). Oceanology 2012; (52)1:79–87.

[16] Sjare BL, Smith TG. The relationship between behavioral activity and underwater vocalizations of the white whale, Delphinapterus leucas. Can J Zool 1986; 64:2824–2831.

[17] Miller LA, Pristed J, Moshl B, Surlykke A. The click-sounds of narwhals (Monodon monoceros) in Inglefield Bay, Northwest Greenland. Mar Mamm Sci 1995; 11:491–502.

[18] Johnson M, Madsen PT, Zimmer WMX, Aguilar de Soto N, Tyack PL. Beaked whales echolocate on prey. Biol. Lett. 2004; 271:383–386. doi: 10.1098/rsbl.2004.0208 15801582 PMC1810096

[19] Nuuttila HK, Meier R, Evans PGH, Turner JR, Bennell JD, Hiddink JG. Identifying Foraging Behaviour of Wild Bottlenose Dolphins (Tursiops truncatus) and Harbour Porpoises (Phocoena phocoena) with Static Acoustic Dataloggers. Aquat Mammal 2013; 39:147–161.

[20] Arranz P, DeRuiter SL, Stimpert AK, Neves S, Friedlaender AS, Goldbogen JA. Discrimination of fast click-series produced by tagged Risso’s dolphins (Grampus griseus) for echolocation or communication. J Exp Biol 2016; 219:2898–2907. doi: 10.1242/jeb.144295 27401759

[21] Herzing DL. Vocalizations and associated underwater behaviour of free-ranging Atlantic spotted dolphin (Stenella frontalis) and bottlenose dolphins (Tursiops truncatus). Aquat Mammal 1996; 22:61–79.

[22] Lammers MO, Au WWL, Aubauer R., Nachtigall PE. A comparative analysis of echolocation and burst-pulse click trains in Stenella longirostris. In: Thomas J, Moss C, Vater M, editors. Echolocation in Bats and Dolphins. Chicago: University of Chicago; 2003. pp. 414–419.

[23] Marrero Pérez J, Jensen FH, Rojano-Doñate L, Aguilar de Soto N. Different modes of acoustic communication in deep-diving short-finned pilot whales (Globicephala macrorhynchus). Mar Mamm Sci 2017; 33:59–79.

[24] Wright BM, Ford JKB, Ellis GM, Deecke VB, Shapiro AD., Battaile BC, et al. Fine-scale foraging movements by fish-eating killer whales (Orcinus orca) relate to the vertical distributions and escape responses of salmonid prey (Oncorhynchus spp.). Movement Ecol 2017; 5(3):1–18. doi: 10.1186/s40462-017-0094-0 28239473 PMC5319153

[25] Holt MM, Hanson MB, Emmons CK, Haas DK, Giles DA, Hogan JT. Sounds associated with foraging and prey capture in individual fish-eating killer whales. Orcinus orca. J Acoust Soc Am 2019; 146:3475–3486. doi: 10.1121/1.5133388 31795684

[26] Shelden KEW, Goetz KT, Rugh DJ, Calkins DG, Mahoney BA, Hobbs RC. Spatio-temporal Changes in Beluga Whale, Delphinapterus leucas, Distribution: Results from Aerial Surveys (1977–2014), Opportunistic Sightings (1975–2014), and Satellite Tagging (1999–2003) in Cook Inlet, Alaska. Mar Fish Rev 2015; 77(2):1–31.

[27] Lowry LF, Frost KJ, Zerbini A, DeMaster D, Reeves RR. Trend in aerial counts of beluga or white whales (Delphinapterus leucas) in Bristol Bay, Alaska, 1993–2005. J Cetacean Res Manage 2008; 10:201–207.

[28] Goetz KT, Montgomery RA, Hoef JMV, Hobbs RC, Johnson DS. Identifying essential summer habitat of the endangered beluga whale Delphinapterus leucas in Cook Inlet, Alaska. Endanger Species Res 2012; 16:135–147.

[29] Castellote M, Small R, Lammers MO, Jenniges J, Mondragon J, Atkinson S. Dual instrument passive acoustic monitoring of belugas in Cook Inlet, Alaska. J Acous Soc Amer 2016; 139:2697–2707. doi: 10.1121/1.4947427 27250163

[30] Castellote M, Small RJ, Lammers MO, Jenniges J, Mondragon J, Garner C, et al. Seasonal Distribution and Foraging Occurrence of Cook Inlet Beluga Whales Based on Passive Acoustic Monitoring. Endang Species Res 2020; 41:225–243.

[31] Martin AR, Smith TG. Deep diving in wild, free-ranging beluga whales, Delphinapterus leucas. Can J Fish Aqua. Sci 1992; 49:462–466.

[32] Norman SA, Goertz CE, Burek KA, Quakenbush LT, Cornick LA, Romano TA, et al. Seasonal hematology and serum chemistry of wild beluga whales (Delphinapterus leucas) in Bristol Bay, Alaska, USA. J Wildl Dis 2012; 48(1):21–32. doi: 10.7589/0090-3558-48.1.21 22247370

[33] Citta JJ, Quakenbush LT, Frost KJ, Lowry L, Hobbs RC, Aderman H. Movements of beluga whales (Delphinapterus leucas) in Bristol Bay, Alaska. Mar Mamm Sci 2016; 32(4):1272–1298.

[34] Johnson MP, Tyack PL. A digital acoustic recording tag for measuring the response of wild marine mammals to sound. IEEE J Oceanic Eng 2003; 28,:3–12.

[35] Lopez R, Malardé JP, Royer F, Gaspar P. Improving Argos Doppler Location Using Multiple-Model Kalman Filtering. IEEE T Geosci Remote 2014; 52:4744–4755.

[36] Andrews RD. 1998. Remotely releasable instruments for monitoring the foraging behaviour of pinnipeds. Mar Ecol Prog Series 1998; 175:289–294.

[37] Lee O, Andrews RD, Burkanov VN, Davis RW. Ontogeny of early diving and foraging behavior of northern fur seal (Callorhinus ursinus) pups from Bering Island, Russia. Mar. Biol. 2014; 161:1165–1178.

[38] Miller PJO, Johnson M, Tyack PL. Sperm whale behavior indicates the use of echolocation click buzzes ‘creaks’ in prey capture. Proc R Soc B Biol Sci 2004; 271:2239–2247. doi: 10.1098/rspb.2004.2863 15539349 PMC1691849

[39] Aguilar Soto N, Madsen PT, Tyack P, Arranz P, Marrero J, Fais A. et al. No shallow talk: cryptic strategy in the vocal communication of Blainville’s beaked whales. Mar Mamm Sci 2011; 28:75–92.

[40] Tennessen JB, Holt MM, Hanson MB, Candice KE, Deborah AG, Hogan JT. Kinematic signatures of prey capture from archival tags reveal sex differences in killer whale foraging activity. J Exp Biol 2019; 222:191874. doi: 10.1242/jeb.191874 30718292

[41] Zimmer WMX, Johnson M, Madsen PT, Tyack PT. Echolocation clicks of free-ranging Cuvier’s beaked whales (Ziphius cavirostris). J Acoust Soc Am 2005; 117:3919–3927. doi: 10.1121/1.1910225 16018493

[42] Johnson M, Madsen PT, Zimmer WMX, Aguilar de Soto N, Tyack PL. Foraging Blainville’s beaked whales (Mesoplodon densirostris) produce distinct click types matched to different phases of echolocation. J Exp Biol 2006; 209:5038–5050. doi: 10.1242/jeb.02596 17142692

[43] Wu-Jung L, Falk B. Callmark: A simple GUI tool for marking animal vocalization in recordings. (Version v1.1); 2021. Database: Zenodo [internet]. Available from: https://zenodo.org/record/4815171#.YTqIXZ2pFhg. doi:

[44] R Core Team. R: A language and environment for statistical computing. R Foundation for Statistical Computing. 2021. Available from: https://www.R-project.org/.

[45] Kaufman L, Rousseeuw PJ. Finding Groups in Data: An Introduction to Cluster Analysis. Wiley, New York; 1992.

[46] Maechler M, Rousseeuw P, Struyf A, Hubert M, Hornik K. Cluster: Cluster Analysis Basics and Extensions. R package version 2.1.2; 2021. Available from: https://CRAN.R-project.org/package=cluster.

[47] Fowlkes EB, Mallows CL. A Method for Comparing Two Hierarchical Clusterings. J Am Stat Assoc 1983; 78(383):553.

[48] Galili T. Dendextend: an R package for visualizing, adjusting, and comparing trees of hierarchical clustering. Bioinformatics. 2015; 31(22):3718–20 doi: 10.1093/bioinformatics/btv428 26209431 PMC4817050

[49] Therneau T, Atkinson B, Ripley B. Recursive partitioning for classification, regression and survival trees (rpart); 2021. Available from: https://cran.r-project.org/package=rpart.

[50] Lammers MO, Castellote M, Small RJ, Atkinson S, Jenniges JJ, Rosinski A. Passive acoustic monitoring of Cook Inlet beluga whales (Delphinapterus leucas). J Acoust Soc Am 2013; 134:2497–2504. doi: 10.1121/1.4816575 23968047

[51] Castellote M, Small RJ, Lammers MO, Jenniges J, Mondragon J, Garner CD, et al. Seasonal Distribution and Foraging Occurrence of Cook Inlet Beluga Whales Based on Passive Acoustic Monitoring. Endang Species Res 202; 41:225–243.

[52] Small RJ, Brost B, Hooten M, Castellote M, Mondragon J. Potential for spatial displacement of Cook Inlet beluga whales by anthropogenic noise in critical habitat. Endang Species Res 2017; 32:43–57.

[53] Goetz KT, Rugh DJ, Read AJ, Hobbs RC. Habitat use in a marine ecosystem: beluga whales Delphinapterus leucas in Cook Inlet, Alaska. Mar Ecol Prog Ser 2007; 330:247–256.

[54] Rugh DJ, Shelden KEW, Hobbs RC. Range contraction in a beluga whale population. Endanger Species Res 2010; 12:69–75.

[55] McGuire TL, Himes Boor GK, McClung JR, Stephens AD, Garner C, Shelden KEW. et al. Distribution and habitat use by endangered Cook Inlet beluga whales: patterns observed during a photo-identification study 2005–2017. Aquat Conserv Mar Freshw Ecosyst 2020; 30(12):2402–2427.

[56] Tregenza N. CPOD.exe: a guide for users. Chelonia Ltd; 2014. Available from: https://www.chelonia.co.uk/cpod_downloads.htm

[57] Koschinski S, Diederichs A, Amundin M. Click train patterns of free-ranging harbour porpoises acquired using T-PODs may be useful as indicators of their behaviour. J Cetacean Res Manag 2008; 10:147−155.

[58] Roy N, Simard Y, Gervaise C. 3D tracking of foraging belugas from their clicks: experiment from a coastal hydrophone array. Appl Acoust 2010; 71:1050−1056.

[59] Zhong M, Castellote M, Dodhia R, Lavista Ferres J, Keogh M, Brewer A. Beluga whale acoustic signal classification using deep learning neural network models. J Acoust Soc Am. 2020; 147(3):1834–1841. doi: 10.1121/10.0000921 32237822

[60] Yanusz R, Merizon R, Evans D, Willette M, Spencer T, Raborn S. Inriver abundance and distribution of spawning Susitna River sockeye salmon Oncorhynchus nerka, 2006. Alaska Department of Fish and Game, Fishery Data Series No. 07–83, Anchorage. 2007. Available from: https://www.adfg.alaska.gov/FedAidpdfs/FDS11-12.pdf

[61] Yanusz RJ, Cleary PM, Campbell J, Horner-Neufeld G, Reed D, DeCovich NA. Abundance, distribution, and surveys of spawning Chinook salmon 2012–2014 and spawning coho salmon 2013–2014 in the Susitna River. Alaska Department of Fish and Game, Fishery Data Series No. 18–16, Anchorage. 2018. Available from: https://www.adfg.alaska.gov/FedAidPDFs/fds18-16.pdf

[62] Quinn PT. The Behavior and Ecology of Pacific Salmon and Trout. 2nd ed. Seattle: University of Washington Press; 2018.

[63] Consensus Building Institute (CBI). Cook Inlet Beluga Whale Research Methods Workshop. Nov. 29–30, 2017, Anchorage, AK. Workshop report prepared for the Alaska Regional Office, NMFS, NOAA; 107 p. Availble from: https://media.fisheries.noaa.gov/dam-migration/cib-research-methods-workshop-report1117.pdf

[64] Akamatsu T, Wang D, Wang K, Li S, Dong S. Scanning sonar of rolling porpoises during prey capture dives. J Exp Biol 2010; 213:146–152. doi: 10.1242/jeb.037655 20008371

[65] Griffin DR, Webster FA, Michael CR. The echolocation of flying insects by bats. Anim Behav.1960; 8: 141–154.

[66] Au WWL. The sonar of dolphins. New York: Springer. 1993

[67] Au WWL, Benoit-Bird KJ. Automatic gain control in the echolocation system of dolphins. Nature 2003; 423:861–863. doi: 10.1038/nature01727 12815429

[68] Madsen PT, Johnson M, Aguilar de Soto N, Zimmer WMX, Tyack P. Biosonar performance of foraging beaked whales (Mesoplodon densirostris). J Exp Biol 2005; 208:181–194. doi: 10.1242/jeb.01327 15634839

[69] Beedholm K, Miller LA. Automatic gain control in harbour porpoises (Phocoena phocoena)? Central versus peripheral mechanisms. Aquat Mamm 2007; 33:69–75.

[70] Atem A, Rasmussen MH, Wahlberg M, Petersen HC, Miller L. Changes in click source levels with distance to targets: studies of free-ranging whitebeaked dolphins (Lagenorhynchus albirostris) and captive harbor porpoises (Phocoena phocoena). Bioacoustics 2009; 19:49–65.

[71] Jensen FH, Bejder L, Wahlberg M. Madsen PT. Biosonar adjustments to target range of echolocating bottlenose dolphins (Tursiops sp.) in the wild. J Exp Biol 2009; 212, 1078–1086. doi: 10.1242/jeb.025619 19329740

[72] Ridgway SH, Moore PW, Carder DA, Romano TA. Forward shift of feeding buzz components of dolphins and belugas during associative learning reveals a likely connection to reward expectation, pleasure and brain dopamine activation. J Exp Biol 2014; 217:2910–2919. doi: 10.1242/jeb.100511 25122919

[73] Ridgway S, Dibble DS, Van Alstyne K, Price D. On doing two things at once: Dolphin brain and nose coordinate sonar clicks, buzzes and emotional squeals with social sounds during fish capture. J Exp Biol 2015; 218:3987–3995. doi: 10.1242/jeb.130559 26567354

[74] Rasmussen MH, Koblitz JC, Laidre KL. Buzzes and high frequency broad band clicks recorded from narwhals (Monodon monoceros) at their wintering feeding ground. Aquat Mammals 2015; 41(3):256–264.

[75] DeRuiter SL, Bahr A, Blanchet MA, Hansen SF, Kristensen JK, Madsen PT. Acoustic behavior of echolocating porpoises during prey capture. J Exp Biol 2009; 212:3100–3107. doi: 10.1242/jeb.030825 19749102

[76] Wisniewska DM, Johnson M, Nachtigall PE, Madsen PT. Buzzing during biosonar-based interception of prey in the delphinids Tursiops truncatus and Pseudorca crassidens. J Exp Biol 2014; 217:4279–4282. doi: 10.1242/jeb.113415 25394631

[77] Au WW, Penner RH, Turl CW. Propagation of beluga echolocation signals. J Acoust Soc Am 1987; 82(3):807–13. doi: 10.1121/1.395278 3655114

[78] Lammers MO, Castellote M. The beluga whale (Delphinapterus leucas) produces two pulses to convolve its echolocation click. Biol Lett 2009; 5(3):297–301. doi: 10.1098/rsbl.2008.0782 19324643 PMC2679917

[79] Aoki K, Amano M, Mori K, Kourogi A, Kubodera T, Miyazaki N. Active hunting by deep-diving sperm whales: 3D dive profiles and maneuvers during bursts of speed. Mar Ecol Prog Ser 2012; 444:289–301.

[80] Woodward BL, Winn JP. Apparent lateralized behavior in gray whales feeding off the central British Columbia coast. Mar Mammal Sci 2006; 22:64–73.

[81] Stimpert AK, Wiley DN, Au WWL, Johnson MP, Arsenault R. ‘Megapclicks’: acoustic click trains and buzzes produced during night-time foraging of humpback whales (Megaptera novaeangliae). Biol Lett 2007; 3:467–470. doi: 10.1098/rsbl.2007.0281 17686753 PMC2391189

[82] Stimpert A, De Ruiter SL, Southall B, Moretti D, Falcone E, Goldbogen J, et al. Acoustic and foraging behavior of a Baird’s beaked whale, Berardius bairdii, exposed to simulated sonar. Sci Rep 2014; 4:7031. doi: 10.1038/srep07031 25391309 PMC4229675

[83] Goldbogen JA, Calambokidis J, Friedlaender AS, Francis J, DeRuiter SL, Stimpert AK, et al. Underwater acrobatics by the world’s largest predator: 360° rolling manoeuvres by lungefeeding blue whales. Biol Lett 2013; 9:20120986. doi: 10.1098/rsbl.2012.0986 23193050 PMC3565519

[84] Cade DE, Friedlaender AS, Calambokidis J, Goldbogen JA. Kinematic diversity in rorqual whale feeding mechanisms. Curr Biol 2016; 26:2617–2624. doi: 10.1016/j.cub.2016.07.037 27666966

[85] Kane EA, Marshall CD. Comparative feeding kinematics and performance of odontocetes: belugas, Pacific white-sided dolphins and long-finned pilot whales. J Exp Biol 2009; 212:3939–3950 doi: 10.1242/jeb.034686 19946072

[86] Wolf N, Harris BP, Richard N, Suresh AS, Lomac-MacNair K, Parker L. High-frequency aerial surveys inform the seasonal distribution of Cook Inlet beluga whales. Wildl Soc Bull 2018; 42:577–586.

[87] Shields P, Frothingham A. Upper Cook Inlet commercial fisheries annual management report, 2017. Alaska Department of Fish and Game, Fishery Management Report No. 18–10, Anchorage. 2018. Available from: http://www.adfg.alaska.gov/FedAidPDFs/FMR19-25.pdf

[88] Alaska Department of Fish and Game (ADF&G). Susitna Hydro aquatic studies, phase II basic data report. Volume 4: Aquatic habitat and instream flow studies, 1982, parts I and II. Alaska Department of Fish and Game Susitna Hydro Aquatic Studies. 447 pp. 1983. Available from: https://www.arlis.org/docs/vol1/Susitna/5/APA585.pdf

[89] Barrett BM, Thompson FM, Wick SN. (1984). Adult anadromous fish investigations: May-October 1983. Susitna Hydro Aquatic Studies, report No. 1. APA Document No.1450. Anchorage: Alaska Department of Fish and Game. 1984. Available from: https://www.arlis.org/docs/vol1/Susitna/14/APA1450.pdf

[90] Gauthier S, Horne JK. Acoustic characteristics of forage fish species in the Gulf of Alaska and Bering Sea based on Kirchhoff-approximation models. Can J Fish Aquat Sci 2004; 61:1839–1850.

