# Supplementary figures and images for: Beluga whale (Delphinapterus leucas) acoustic foraging behavior and applications for long term monitoring
Source: PLoS One. 2021 Nov 30;16(11):e0260485. doi: 10.1371/journal.pone.0260485 (PMC8631677; doi:10.1371/journal.pone.0260485)

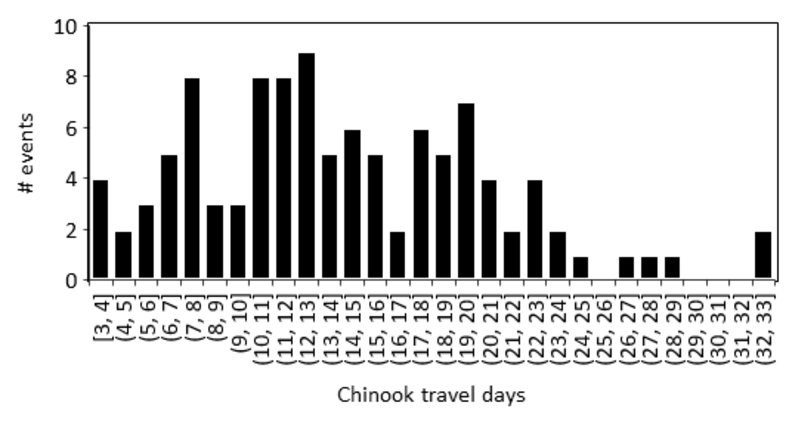

Supplement: S1 Fig — (TIF) [file pone.0260485.s001.tif]
